# Lymphatic Reconstruction in Kidney Allograft Aggravates Chronic Rejection by Promoting Alloantigen Presentation

**DOI:** 10.3389/fimmu.2021.796260

**Published:** 2021-12-09

**Authors:** Jinwen Lin, Ying Chen, Huijuan Zhu, Kai Cheng, Huiping Wang, Xianping Yu, Mengmeng Tang, Jianghua Chen

**Affiliations:** ^1^ Kidney Disease Center, The First Affiliated Hospital, College of Medicine, Zhejiang University, Hangzhou, China; ^2^ Department of Pathology, The First Affiliated Hospital, College of Medicine, Zhejiang University, Hangzhou, China; ^3^ Xiangya School of Medicine, Central South University, Changsha, China

**Keywords:** renal transplantation, chronic rejection, inflammation, lymphangiogenesis, allograft

## Abstract

Chronic rejection of the renal allograft remains a major cause of graft loss. Here, we demonstrated that the remodeling of lymphatic vessels (LVs) after their broken during transplantation contributes to the antigen presenting and lymph nodes activating. Our studies observed a rebuilt of interrupted lymph draining one week after mouse kidney transplantation, involving preexisting lymphatic endothelial cells (LECs) from both the donor and recipient. These expanding LVs also release C-C chemokine ligand 21 (CCL21) and recruit CCR7^+^ cells, mainly dendritic cells (DCs), toward lymph nodes and spleen, evoking the adaptive response. This rejection could be relieved by LYVE-1 specific LVs knockout or CCR7 migration inhibition in mouse model. Moreover, in retrospective analysis, posttransplant patients exhibiting higher area density of LVs presented with lower eGFR, severe serum creatinine and proteinuria, and greater interstitial fibrosis. These results reveal a rebuilt pathway for alloantigen trafficking and lymphocytes activation, providing strategies to alleviate chronic transplantation rejection.

**Graphical Abstract:**
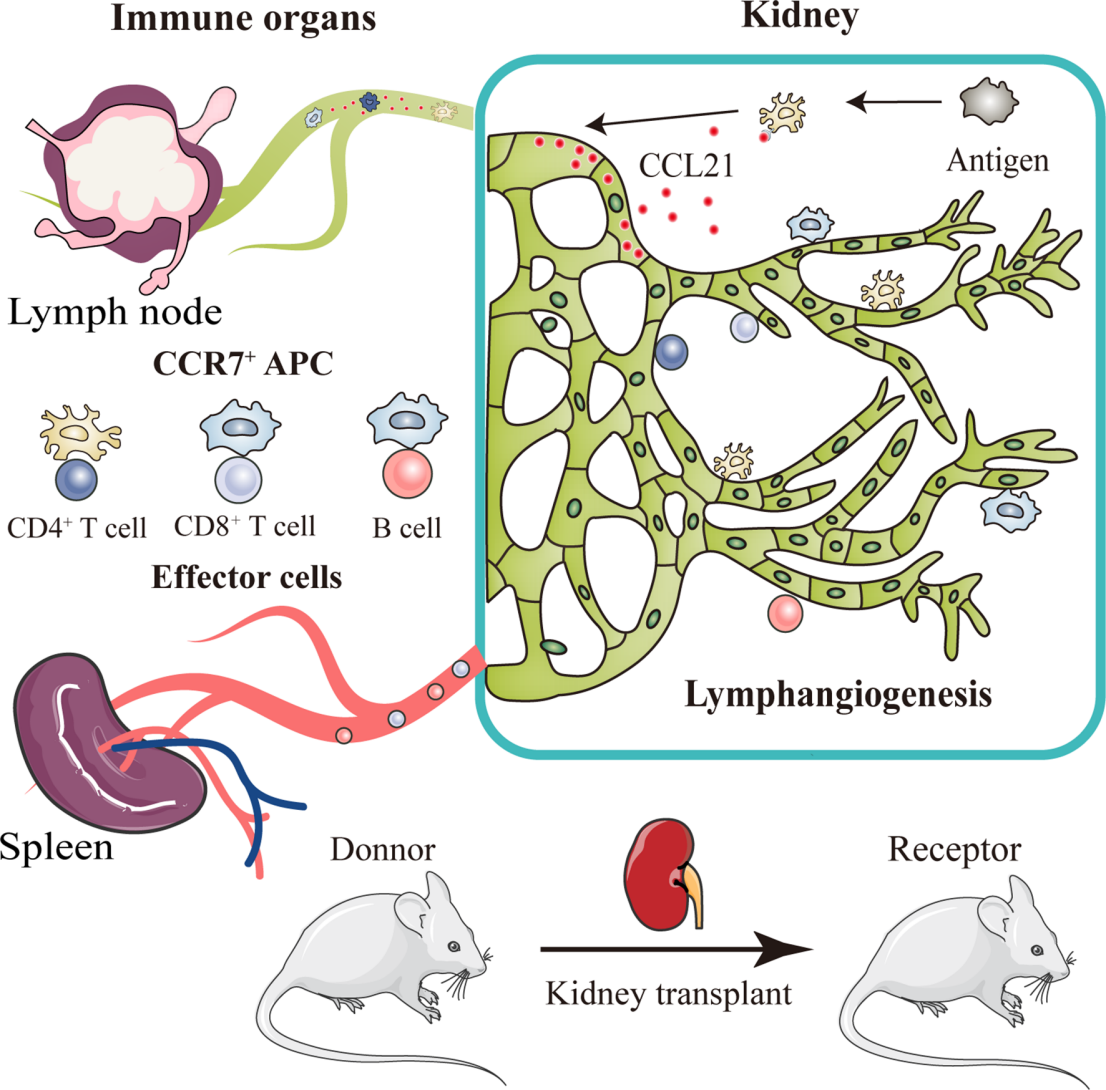
After kidney transplantation, a triad crosstalk of CCR7-expressing antigen presenting cells (APCs), CCL21-expressing lymphatic vessels, and effector lymphocytes was demonstrated, which promote alloimmune response. Injured lymphatic endothelial cells release CCL21, guiding the migration of CCR7^+^ APCs to adjacent lymph nodes through broken lymphatic vessels. This migration then activates effector lymphocytes and aggravate lymphangiogenesis, leading to more severer organ rejection.

## Introduction

Chronic allograft rejection is a curial contributor to allograft failure, and this adaptive immunity is generally supposed to be the activation against HLA molecules aroused by donor passenger leukocytes (DPLs) or recipient antigen-presenting cells (APCs) (1). These messenger cells mobilize from graft to the secondary lymphoid organs, linking the regional immunity with a larger immune system and playing a pivotal role in antigen-mediated rejection. However, as the predominant initial process, how, and where the donor antigens are presented to adaptative immunity and caused the allograft damage remains unclear, given the trafficking conduit formed by LVs is broken intraoperatively.

In non-transplant models, studies have demonstrated that this sensitization was by detecting immune complex on dendritic cells (DCs), follicular DCs, or certain macrophages (2–4). The prevailing key of this guiding pathway has been that these APCs expressing CCR7 are guided by CCL21, a chemokine released by lymphatic endothelial cells (LECs), and mobilize from *in situ* to lymph nodes. However, in patients undergoing renal transplantation, the arteries, veins, and lymphatic vessels (LVs) are all broken, while only the arteries and veins are connected post-surgery, resulting the interrupted lymph drainage (5, 6). Correspondingly, DPLs, especially DCs were rarely observed in host draining lymph nodes 4-7 days after transplantation, indicating the broken of lymph draining pathway (7, 8). Alternative routes of doner antigen-presenting pathways have also been proposed: (1) reverse transmigration in blood circulation, (2) forming ectopic lymphoid organ in allograft, and (3) extracellular vesicles presenting (9). However, the first possibility is in rare case of reverse transmigration from tissue directly into blood vessels (10). Nevertheless, the ectopic lymphoid organ and extracellular vesicles format, though have been reported, the relative uncommon phenomenon couldn’t explain the prevalence and severity of chronic rejection (11). Therefore, understanding the underlying mechanism of antigen-presenting in transplantation and if an unreported pathway plays a role are critical for elucidating how chronic rejection is shaped after transplantation.

Previous studies have revealed that lymphangiogenesis can be promoted under various inflammatory environment resulting from transplantation and other injuries (12). The exposure of vascular endothelial growth factor receptor 3 (VEGFR3) to vascular endothelial growth factor C (VEGF-C) and VEGF-D has been widely demonstrated as the pivotal molecular event in lymphangiogenesis in unilateral ureteral obstruction (UUO), proteinuria and hypertension caused renal inflammation (13–15). Besides, some fibrogenic cytokines like fibroblast growth factor-2 (FGF-2), transforming growth factor-β1 and connective tissue growth factor have also been verified the effects on lymphangiogenesis in UUO model (13, 16, 17). In renal transplantation, accumulating biopsies demonstrate obvious lymphatic neoangiogenesis in allograft which strongly correlates with inflammatory lymphocytic infiltration (18, 19). However, in condition of lymphatic destruction, the underlying mechanisms of lymphangiogenesis and importantly the consequences of lymphatic drainage are largely unclear. Lymphatic restore and increasing lymphatic flow from the donor graft to draining lymph nodes have been revealed to promote immune trafficking after transplantation of solid organs including heart and lung (20, 21). Increasing spread of lymphatic chemokines in kidney allograft was shown to enhance the recruitment of antigen presenting cells towards the lymphatic vessels (22, 23). Inhibited antigen presentation was supposed to be closely related with the production of donor-specific antibody (DSA). This way, lymphangiogenesis is, perhaps, a powerful enabler to the production of DSA and DSA mediated allograft rejection. By contrast, the mechanisms by which lymphatic vessels reconnect in renal allograft, and if the chemical gradient of CCR7-CCL21 are involved, remain unknown. In addition, stimulation of lymphangiogenesis was also found to accelerate antigen clearing and inhibit chronic skin inflammation, embodying multiple roles in both initiation and resolution of immune responses upon specific regional microenvironment (24).

To date, the anastomosis of donor-host LVs and the mechanisms of alloantigen presenting by remodeled LVs post-operation have not, to our knowledge, been answered in chronic renal rejection. Here, we demonstrated that the reconnection of LVs after their broken during transplantation contributes to the antigen presenting and lymph nodes activating, utilizing the LVs reporter system in a murine model of chronic renal rejection. Our studies observed obvious lymphangiogenesis and a rebuilt of interrupted lymph draining one week after surgery, involving preexisting LECs from both the donor and recipient. These expanding LVs released CCL21 and recruited CCR7^+^ cells, mainly DCs, toward lymph nodes and spleen, resulting the adaptive response. This rejection could be relieved by LYVE-1 specific LVs knockout, or CCR7 migration inhibition. Moreover, in our retrospective analysis, posttransplant patients exhibiting higher area density of LVs presented with lower eGFR, severe serum creatinine and proteinuria, and greater intrarenal interstitial fibrosis indicating a chronic decrease in renal function. These findings identify a novel pathway of doner alloantigen presenting through restoring the broken lymph flow and add to our understanding of the complex regulation of chronic rejection.

## Results

### Chronic Rejection Is Associated With Lymphangiogenesis in Renal Allograft

**Figure 1:**
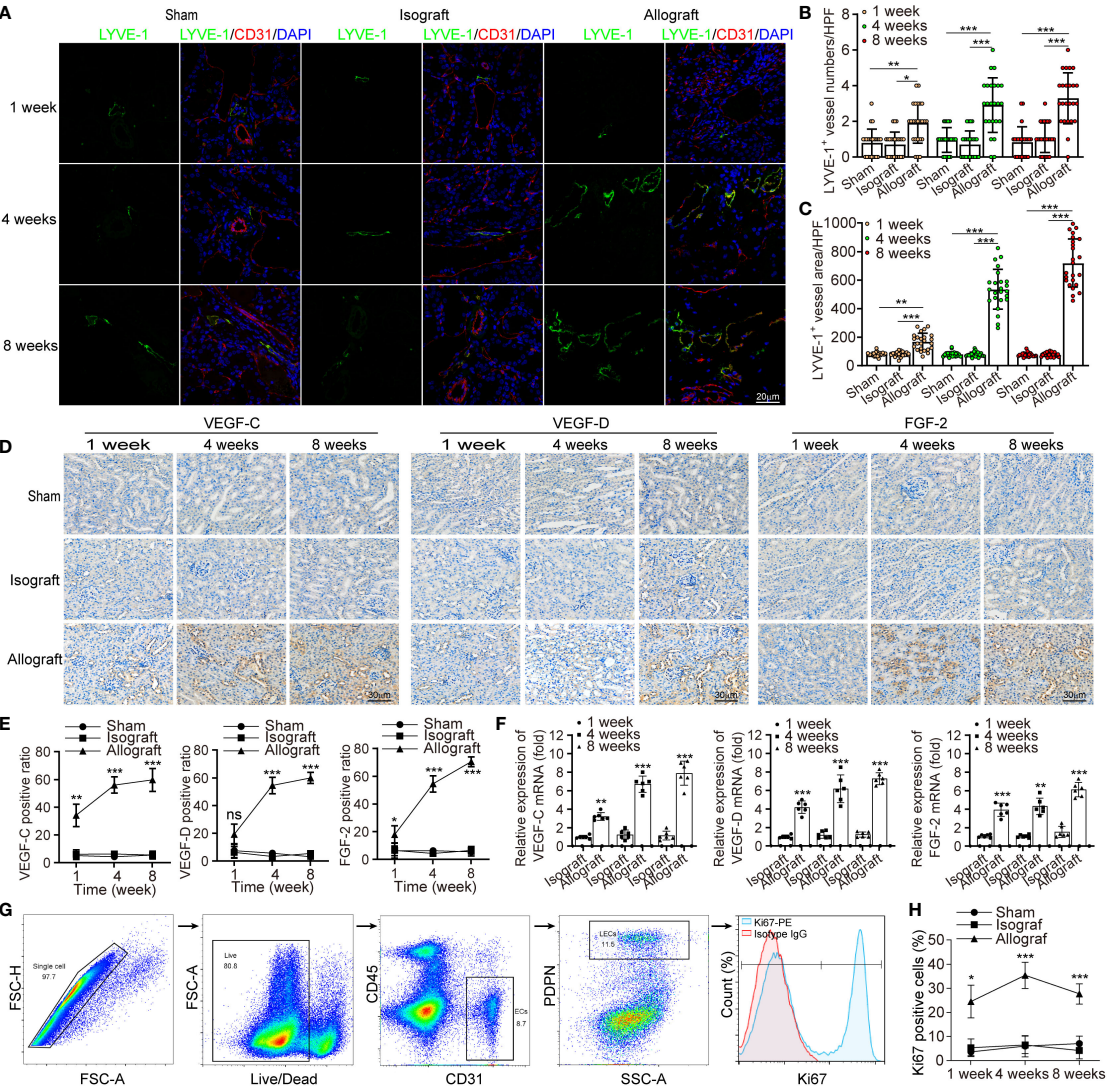
Chronic rejection is associated with lymphangiogenesis in renal allograft. **(a)** Representative immunofluorescence images of LYVE-1, CD31, and DAPI within sham (n=6 mice), isograft (n=6 mice) and allograft (n=6 mice) kidneys at 1, 4 and 8 weeks respectively. **(b and c)** Numbers and area counting of LYVE-1+ vessels in high-power field (HPF) at 1, 4 and 8 weeks respectively. (d) Immunohistochemistry of VEGF-C, VEGF-D and FGF-2 expression within sham, isograft and allograft kidneys at 1, 4 and 8 weeks respectively. **(e)** Positive ratio of VEGF-C, VEGF-D and FGF-2 within sham, isograft and allograft kidneys at 1, 4 and 8 weeks respectively. (f) Relative mRNA expression of VEGF-C, VEGF-D and FGF-2 by qRT-PCR within isograft and allograft kidneys at 1, 4 and 8 weeks respectively, using sham as a reference. **(g)** Live single CD45- PDPN+ CD31+ LECs isolating from renal allograft by gating technology via flow cytometry, and the population of Ki67+ cells. **(h)** The ratio of Ki67+ cells in PDPN+CD31+ LECs within sham, isograft and allograft kidneys at 1, 4 and 8 weeks respectively. *P < 0.05, **P < 0.01, ***P < 0.001. Values are mean ± SEM.

To characterize the intrarenal lymphangiogenesis in kidney allograft, we used Balb/c (H-2d) as the donor source and C57BL/6 (H-2b) as the recipient to establish a chronic rejection model to evaluate the pathological features of chronic rejection, such as interstitial fibrosis and tubular atrophy (**Figure S1**). It’s well known that chronic allograft nephropathy (CAN)features with vasculopathy involving the damage of parenchyma, tubules and vasculitis (25). Confocal imaging on thick sections from renal allograft labeling the LECs marker LYVE-1 suggested that the density of LVs significantly increased in the allografts with a mean of 2.92 versus 0.71, and 3.29 versus 1.08 per 24 HPF at week 4 and 8 posttransplant, respectively compared with sham group, but not the isograft group (**Figures 1A, B**). With time, the lymphatic density increased over 3-fold in the renal allografts, from 0.96 to 3.29 at week 8 in comparison with week 1 posttransplant. Furthermore, we counted the area density of LVs which is regarded for better reflecting the flow amount of lymph drainage, and observed an increase at week 4 and 8 in the allografts in comparison with sham (**Figure 1C**, mean of 535.88 μm^2^ versus 80.47 μm^2^, and 719.54 μm^2^ versus 77.77 μm^2^ per 24 HPF at week 4 and 8 posttransplant, respectively). To confirm the specificity of LYVE-1, another LECs marker, VEGFR3 was tested. In line, enhanced enrichment of LVs number and area density pointed at a highly significant lymphangiogenesis in renal allograft (**Figure S2**). These data suggest that active expansion and enrichment of LVs have occurred in the renal allografts under chronic rejection inflammation. In order to characterize the role of allograft microenvironmental factors in the chronic rejection induced lymphangiogenesis, we further measured the protein and transcript levels of some lymphangiogenic growth factors VEGF-C, VEGF-D and FGF-2 as assessed by immuno-histochemistry and qRT-PCR. We observed that these cytokines were all up-regulated in renal tubular epithelial cells and some renal interstitial cells after renal allograft (**Figures 1D**–**F**). With the finding of highly expressed lymphangiogenic growth factors, we would like to validate that LECs were in an active proliferative state. By flow cytometry, we gated LECs on CD31^+^, PDPN^+^ and CD45^-^ cells, and measured the expression level of Ki67 (**Figure 1G**). The results confirmed a significant increase in the percentage of Ki67^+^ mitotically active LECs in renal allografts, compared with the sham and isograft groups (**Figure 1H**), and the proliferation may reach its peak around week 4 with the 35.33 positive ratio of Ki67^+^ cells. Overall, all these data suggest that renal allograft is accompanied by a local lymphangiogenic state and lymphangiogenesis is related to chronic rejection inflammation.

### Lymphangiogenesis in Renal Allograft Accompanied With Inflammatory Cell Infiltration Mediated by CCL21 Expression in LECs

**Figure 2:**
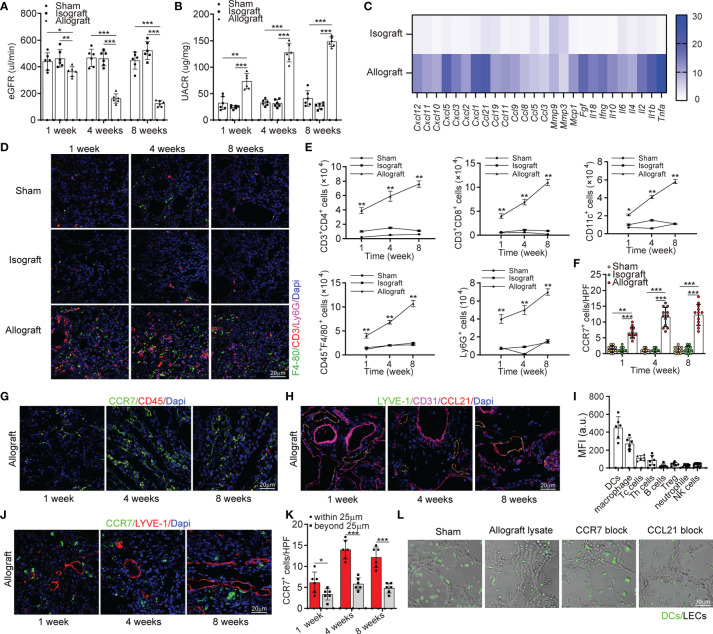
Lymphangiogenesis in renal allograft accompanied with degenerated renal function and inflammatory cell infiltration mediated by CCL21 expression in lymphatic endothelial cells. **(A)** eGFR level in sham (n=6 mice), isograft (n=6 mice), and allograft (n=6 mice) groups at 1, 4 and 8 weeks respectively. **(B)** UACR level in sham, isograft, and allograft groups at 1, 4 and 8 weeks respectively. **(C)** Messenger RNA expression of pro-inflammatory cytokines by multiplex reverse transcription-polymerase chain reaction assay. The heat map displays the relative expression level in isografts and allografts compared to that in the sham control. **(D)** Representative immunofluorescence images showing F4-80^+^, CD3^+^, Ly6G^+^ cells in sham, isograft, and allograft groups at 1, 4 and 8 weeks respectively. **(E)** Numbers of Th cells, macrophages, dendritic cells, B cells, and neutrophile granulocytes in sham, isograft, and allograft groups determined by flow cytometry at 1, 4 and 8 weeks respectively. **(F)** CCR7^+^ cell counting in sham, isograft, and allograft groups at 1, 4 and 8 weeks respectively. **(G)** Immunofluorescence of CCR7^+^, and CD45^+^ cells in allografts at 1, 4 and 8 weeks respectively. **(H)** Representative immunofluorescence images showing LYVE-1^+^, CD31^+^ and CCL21^+^ cells in allografts at 1, 4 and 8 weeks respectively. **(I)** FACS analysis of the different CCR7^+^ cell types in kidney allograft infiltrated CD45^+^ immune cells: DCs (CD45^+^ and CD11c^+^), macrophage (CD11b^+^ and F4/80^+^), cytotoxic T cells (CD8^+^), T helper cells (CD4^+^), B cells (CD19^+^), Treg cells (CD4^+^, CD25^+^ and FOXP3^+^), neutrophils (CD11b^+^ and Ly6G^+^) and NK cells (NK1.1^+^). **(J)** Representative immunofluorescence images showing the distribution of CCR7^+^ cells and LYVE-1^+^ cells in allografts to confirm the chemotaxis at 1, 4 and 8 weeks respectively. **(K)** Cell counting of CCR7^+^ cells that have different distances (within or beyond 25 μm) from lymphatic endothelial cells. **(L)** Representative images of dendritic cells migrating to lymphatic capillary-like structures. All scale bars represent 20 μm. *P < 0.05, **P < 0.01, ***P < 0.001. Values are mean ± SEM.

In order to explore the correlation between lymphangiogenesis and allograft rejection, we investigated renal function of allograft and concomitant lymphangiogenesis after transplantation. In terms of renal function, the allografts presented a lower estimated glomerular filtration rate (eGFR), greater urinary albumin to creatinine ratio (UACR) (**Figures 2A, B**). To characterize the rejection that directly leads to this dysfunction, we measured messenger RNA expression of pro-inflammatory cytokines and confirmed their great up-regulation in allografts than in other groups by multiplex qRT-PCR (**Figure 2C**). Correspondingly, more infiltrated F4-80^+^, CD3^+^, and Ly6G^+^ cells in allografts than other groups were observed by immunofluorescence analysis (**Figure 2D**). Among these infiltrated inflammatory cells, Th cells, DCs, macrophages, and neutrophile granulocytes presented a striking increase within 8 weeks as analyzed by flow cytometry, which could be viewed as the source of up-regulated pro-inflammatory cytokines and lymphangiogenic growth factors (**Figure 2E**). Therefore, these data suggested a reciprocal relationship between lymphangiogenesis and alloimmunity. To further elucidate the driving force of the infiltrating immune cells and the role of lymphangiogenesis in alloimmune response, we studied the cells expressing CCR7 and its ligand CCL21. A dramatic increase of CCR7^+^ cells was observed in the allografts and found CCR7 was expressed by CD45^+^ infiltrating immune cells but not by endothelial or interstitial cells *in situ* (**Figures 2F**–**H**). Following these results, we further characterized CCR7^+^ cells components within allograft by flow cytometry. DCs constituted most of the CCR7^+^ cells population, then macrophages, Tc cells, and Th cells (**Figure 2I**). However, due to the low expression level of CCR7 in T cells and Treg cells, they do not play a dominant role in chemotaxis mediated by CCR7-CCL21. As expected, our immunofluorescence further verified that the chemokines CCL21 was only expressed by LVs but not blood vessels (**Figure 2I**). In order to visually present the function of lymphangiogenesis in recruiting CCR7^+^ cells, we investigated the distribution of CCR7^+^ cells. As the alloimmunity progresses, we found more of them were transmigrating within LVs and adjacent (within a 25 μm distance) to LECs (**Figures 2J, K**, and **S4**). Based on the finding that CCR7-CCL21 interaction contributed to inflammatory cell infiltration in mice, we predicted that the blocking of either CCR7 by using antibodies or CCL21 by siRNA mediated gene silencing could stop this recruitment. So, we used allograft lysate to mimic an inflammatory micro-environment *in vitro*, and in both CCR7 and CCL21 block groups, the chemotaxis of dendritic cells toward LECs was prevented (**Figure 2L**). Meanwhile, the attached DCs number within 5μm and 3μm of lymphatic capillary-like structures diameter in allograft lysate group was obviously higher than that in sham, CCR7 block and CCL21 block groups, which also indicated that CCR7-CCL21 interaction could decrease the DCs to migrated toward LECs (**Figure S5**). Generally, these findings suggest that through the interaction of CCR7 and CCL21, lymphangiogenesis could drive the infiltration of immune cells (especially dendritic cells) in the kidney.

### Determination of the Reconnection and Origin of LVs

**Figure 3:**
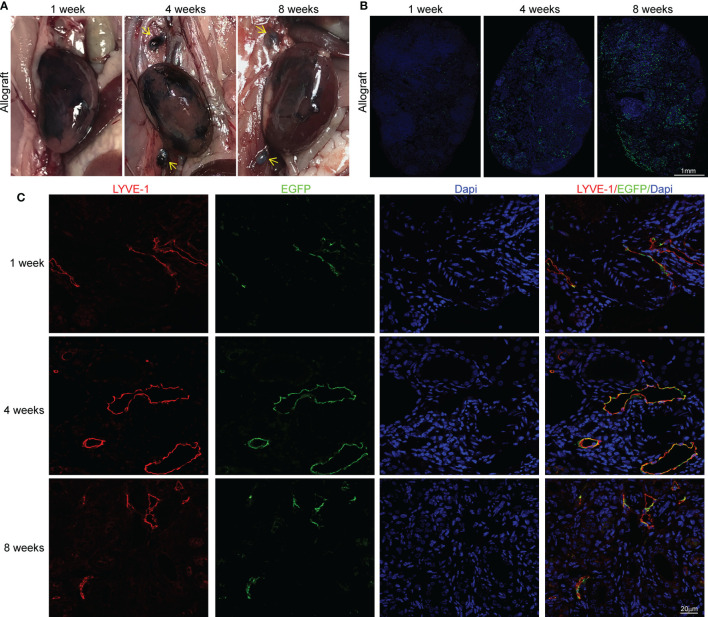
Determination of the reconnection and origin of LVs **(A)** Staining of renal draining lymph nodes (yellow arrows) after the injection of Evans Blue under renal capsule in sham (n=6 mice), isograft (n=6 mice), and allograft (n=6 mice) groups at 1, 4 and 8 weeks respectively. **(B)** Representative immunofluorescence images showing the dendritic cells separated from peripheral blood of Itgax-Cre-GFP mice in renal draining lymph nodes after injection of these cells under the renal capsule at the 1st, 4th, and 8th week after surgery. Scale bar represents 1 mm. **(C)** Co-localization of LYVE-1^+^ and EGFP^+^ cells to explore the donor or recipient origin of lymphatic vessels at 1, 4 and 8 weeks respectively. Scale bar represents 20 μm.

Although we demonstrated the lymphangiogenesis and its interaction with immune cells in allografts, its contribution to alloimmunity cannot be precisely stated unless we clarify how much lymphangiogenesis altered the lymph flow from allograft to renal draining lymph nodes. Therefore, to visualize the lymph draining in allograft, we injected Evans Blue dye solution into the kidney parenchyma after transplantation. The obviously stained lymph nodes in the 4th and 8th week indicate that the lymph flowing to the renal draining lymph nodes was reconstructed at the fourth week at the latest (**Figure 3A**). As DCs constituted most of the CCR7^+^ cells, we then isolated DCs expressing GFP from peripheral blood of Itgax-Cre-GFP mice to study their cellular trafficking by injecting them under renal capsule after the existence of injected DCs in kidney capsule was primarily verified (**Figure S3**). Then we observed their trafficking to the renal draining lymph nodes, this time, some DCs could be observed even after the first week and kept increasing within 8 weeks (**Figure 3B**). Interestingly, there was a time lag between the DCs and the Evans Blue dye reaching the renal draining lymph nodes. This might be because the different distinguishability between the dye, which must be identified visually, and the cells, which could be detected by microscopy. Another explanation for the inconsistent was that CCR7^+^ DCs could be guided by chemical gradient even before the lymph flow was restored, while the dye could only arrive lymph nodes after the reconnection of lymphatic vessels.

After verifying the restoration of lymph drainage after transplantation, we further investigated the anatomical basis for the reconnection of LVs, which is still not thoroughly studied. To allow a visualized analysis of donor or host LVs, donor kidneys were acquired from LYVE-1 EGFP-Cre mice to express EGFP in LVs while cells from host can only express LYVE-1. The co-localization of LYVE-1 and EYFP at the 1st, 4th, and 8th week indicates that cells from the donor and recipient formed physical connections with each other and contributed comparably to the lymphangiogenesis (**Figure 3C**). We further suggest that the restoration of lymph drainage was gained by the progress of heterogeneous sprouting LVs, which reestablished the anastomoses between donor and recipient around the 4th week. Altogether, these findings indicate that the lymph flow from renal to lymph nodes could be regained after transplant with a comparable contribution on the sprouting of LVs from donor and recipient, which finally achieved donor-recipient lymphatic connections.

### Conditional Knockout of LVs Attenuated CCR7^+^ Cells Expansion and Ameliorates Allograft Function

**Figure 4:**
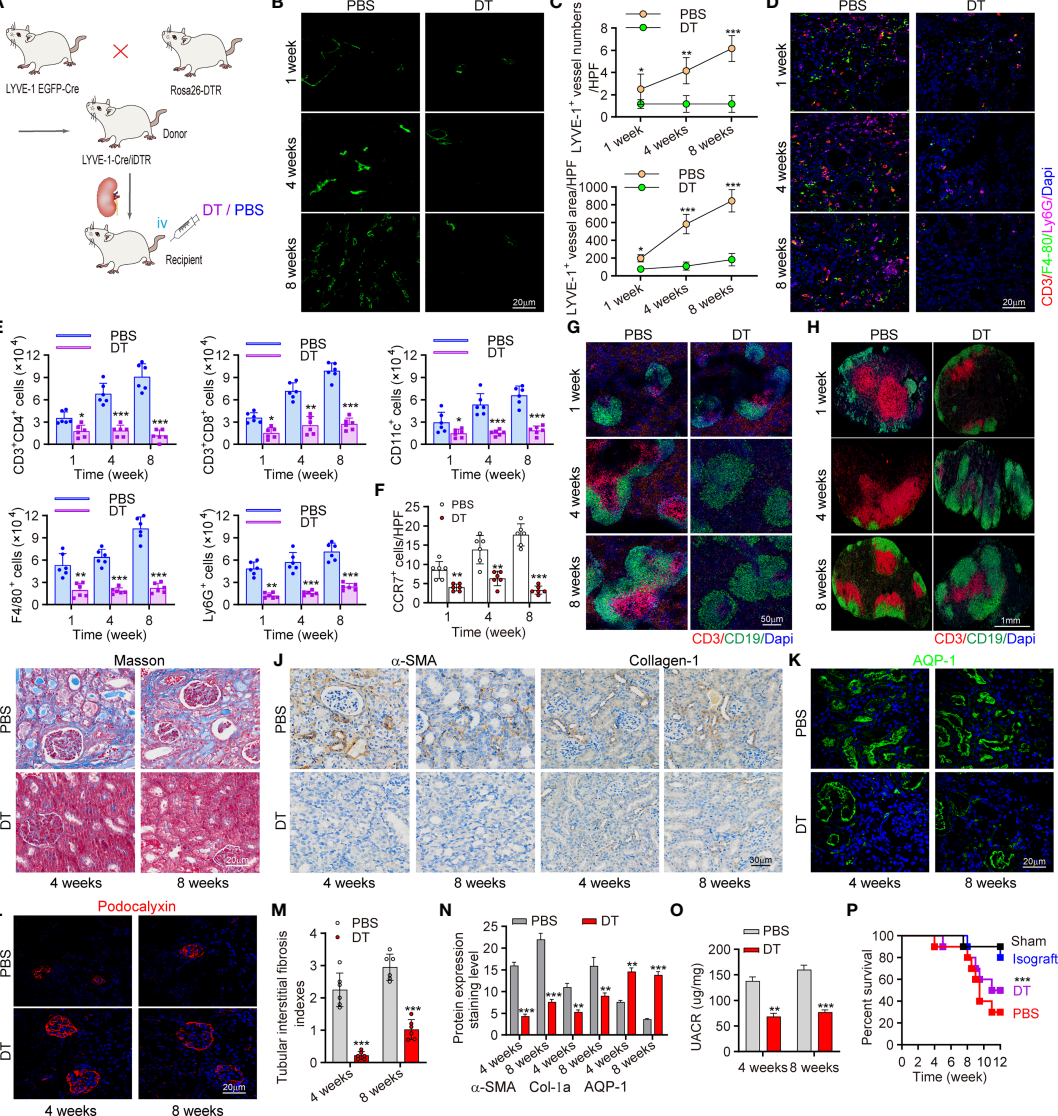
Conditional knockout of LVs attenuated CCR7+ cells expansion and ameliorates allograft function. **(A)** Scheme showing the establishment of conditional LVs KO mouse model and allograft model. Intervention controls: DT (n=6 mice) or PBS (n=6 mice) injection. **(B)** Immunofluorescence of LYVE-1 labeling intrarenal LVs. **(C)** Numbers and area of intrarenal LYVE-1^+^ vessel counted in HPF. **(D)** Representative immunofluorescence images of intrarenal CD3^+^, F4/80^+^ and Ly6G^+^ cells. **(E)** Inflammatory cells infiltration in kidneys analyzed by flow cytometry. **(F)** Visual counting of CCR7^+^ cells with confocal microscopy. **(G, H)** Immunofluorescence of B cells and T cells in spleen (left) and RDLN (right). **(I)** Masson staining of renal tissues. **(J)** The expression of α-SMA and collagen-1 assessed by immunohistochemistry. **(K, L)** Immunofluorescence of AQP-1 and Podocalyxin. **(M)** The assessment of tubular interstitial fibrosis indexes. **(N)** Quantitative analysis of fibrosis–related protein expression. **(O)** Changes of UARC in kidney transplant mice. **(P)** Precent survival of recipient mice in different groups. *P < 0.05, **P < 0.01, ***P < 0.001. Values are mean ± SEM. iv, intravenously; DT, diphtheria toxin; RDLN, renal draining lymph node; AQP, aquaporin-1.

To confirm the role of increased lymph drainage in transplant rejection, we tried to prevent lymph drainage by ablating LVs. The LYVE-1-Cre/inducible DTR (iDTR) double transgenic mouse model was established from LYVE-1 EGFP-Cre×Rosa26-DTR transgenic mice, in which LVs could be selectively ablated following diphtheria toxin (DT) administration (**Figures 4A** and **Figure S4**) (26). DT induced an overt reduction of LVs, as the exact number and area of LYVE-1^+^ vessels decreased by 81.62% and 78.56% versus controls at week 4 and week 8 posttransplant, respectively (**Figures 4B, C**). Next, to characterize the altered cell distribution under the absence of LVs, markers of monocytes, macrophages, and T cells were stained. We found much lower levels of these immune cells with LVs KO, while LYVE-1-Cre didn’t result in any additional effect in controls (**Figure 4D**). Given that macrophages and T cells are main population of CCR7^+^ cells, we further estimated the changes of more CCR7^+^ cells, including CD3^+^CD4^+^ and CD3^+^CD8^+^ T cells, CD11c^+^ DCs, F4/80^+^ macrophages and Ly6G^+^ monocytes as assessed by flow cytometry. Strikingly, all these immune cells decreased by 65.58% and 76.14% at least at week 4 and week 8 posttransplant, respectively with LVs KO (**Figure 4E**). In terms of DSA mediated rejection, the levels of activated B cells, DSA accumulation and C4d deposition were assessed and showed comparative downregulation with DT administration (**Figures S6**, **S7**). In line, the total numbers of CCR7+ cells also showed a 56.31% downtrend (**Figure 4F**). Following the finding that CCL21 in LECs mediated CCR7^+^ cells infiltration, we hypothesized that the interaction of CCR7 and CCL21 was impaired in the absence of LVs, and thus alleviated chronic rejection in renal allograft. In LVs KO mice, spleen, and renal draining lymph node (RDLN) also appear reduced in size and weight compared to controls (**Figure S8**). Immunofluorescence staining suggested that the T cell activation, present lymphoid follicles, and germinal centers were strongly suppressed both in spleen and RDLN (**Figures 4G, H**). Over time, the local inflammation and fibrosis in kidney were alleviated, as LVs KO led to lower levels of α-SMA and collagen-1 labeling renal fibrosis, and the ameliorated atrophy and disconnection of glomerulus was observed by Masson staining. (**Figures 4I, J, N**). Surprisingly, we found that the tubular interstitial fibrosis indexes in allograft was decreased by 74.13% and 56.45% at the week 4 and week 8, respectively versus controls (**Figure 4M**). Along with the improved renal inflammation and fibrosis, renal function also appeared a tremendous difference, as the levels of AQP-1 and Podocalyxin reflecting renal function were both increased. Consistently, with decreased UACR, the strategy of LVs KO effectively enhanced the survival rate of allograft recipient mice compared to PBS group (**Figures 4O, P**). Together, all these data suggested that conditional LVs KO impaired the trafficking of CCR7^+^ cells to LVs, and alleviated kidney inflammation and fibrosis, accompanied by improved kidney functions.

### CCR7 Blocking Antibody Attenuated Lymphocyte Recruitment and Allograft Function Losing

**Figure 5:**
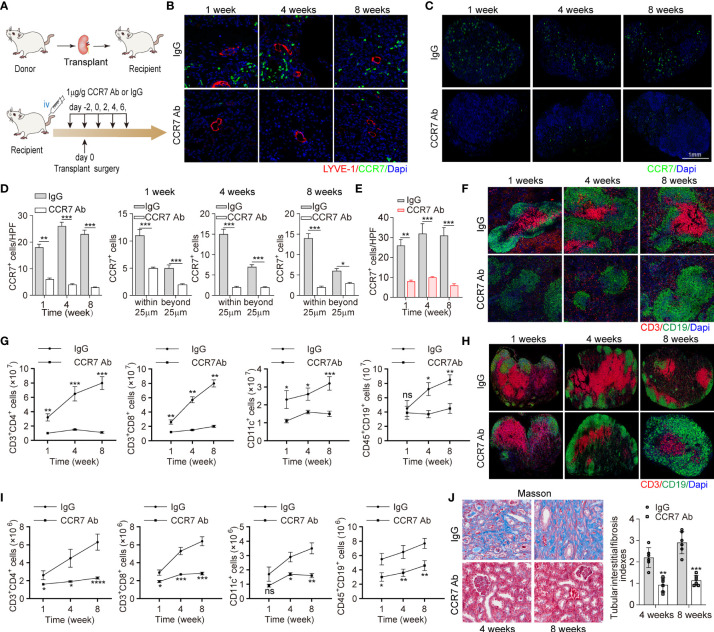
CCR7 blocking antibody attenuated lymphocyte recruitment and allograft function losing. **(A)** Scheme showing recombinant anti-CCR7 antibody intervention in C57BL/6 (H-2b) mouse model (n=6 mice). Control group (n=6 mice): rabbit anti-mouse IgG. **(B)** LYVE-1 and CCR7 staining of kidney tissues following IgG or CCR7 antibody treatment. **(C)** Representative images of CCR7 in RDLN. **(D)** Cell counting of intrarenal CCR7^+^ cells at the 1, 4, and 8 weeks after surgery, including the exact cell counting within or beyond 25 μm respectively. **(E)** Cell counting of CCR7^+^ cells in RDLN. **(F, H)** Immunofluorescence staining of CD3^+^ and CD19^+^ in spleen (upper) and RDLN (bottom). **(G, I)** The numbers of CD3^+^CD4^+^ and CD3^+^CD8^+^ T cells, CD11c^+^DCs and CD45^+^CD19^+^ B cells in spleen (upper) and RDLN (bottom) determined by flow cytometry. **(J)** Masson staining of renal tissues (left), and tubular interstitial fibrosis indexes (right) in recipient mice at the 4 and 8 weeks after surgery. *P < 0.05, **P < 0.01, ***P < 0.001. Values are mean ± SEM.

To further elucidate that suppressing CCR7^+^ cells recruitment ameliorated intrarenal inflammation and fibrosis, CCR7 blocking antibody was administrated to recipient mice (**Figure 5A**). Quantitative analysis revealed that with CCR7 antibody, more than 50% CCR7^+^ cells had decreased and the significant difference was mainly restricted around LVs (within 25μm), which confirmed impaired interaction of CCR7 and CCL21 mediated by CCL21^+^ LECs (**Figures 5B, D**). In line, we observed decreased percentages of various CCR7^+^ cells in RDLNs, indicating that the recruitment of CCR7^+^ cells was still inhibited with CCR7 antibody, even if the lymphangiogenesis was unaffected (**Figures 5C, E**). In addition, we estimated the degree of inflammatory cell infiltration in RDLN and spleen, aiming to figure out the association of CCR7^+^ cells inhibition with systematic inflammation. It’s been observed that CCR7 antibody effectively inhibited the activation of CD3^+^ T cells with reduced germinal centers (**Figures 5F, H**). Meanwhile, the exact changes of major CCR7^+^ cells in RDLN and spleen were measured, including CD3^+^CD4^+^ and CD3^+^CD8^+^ T cells, CD11c^+^ DCs and CD45^+^ CD19^+^ B cells. The strikingly decreased CCR7^+^ cells, activated B cells and DSA levels were observed, which corroborated the suppressed systematic inflammation with CCR7 blockade (**Figures 5G, I** and **Figures S9**, **S13**). To reveal the influence of inhibiting CCR7 ^+^ cells trafficking on allograft, Masson staining of renal tissues was performed, and it’s been found that this strategy effectively ameliorated tubular interstitial fibrosis and renal atrophy at the week 4 and week 8 posttransplant, as well as significantly increased the percent survival of recipient mice, showing similar results as the strategy of suppressing lymphangiogenesis (**Figures 5J**, **S10**). Together, above results elucidated that inhibiting the drainage of CCR7^+^ cells greatly alleviated chronic rejection and renal fibrosis.

### Intrarenal Lymphangiogenesis Negatively Correlated With Allograft Function in Transplant Patients

**Figure 6:**
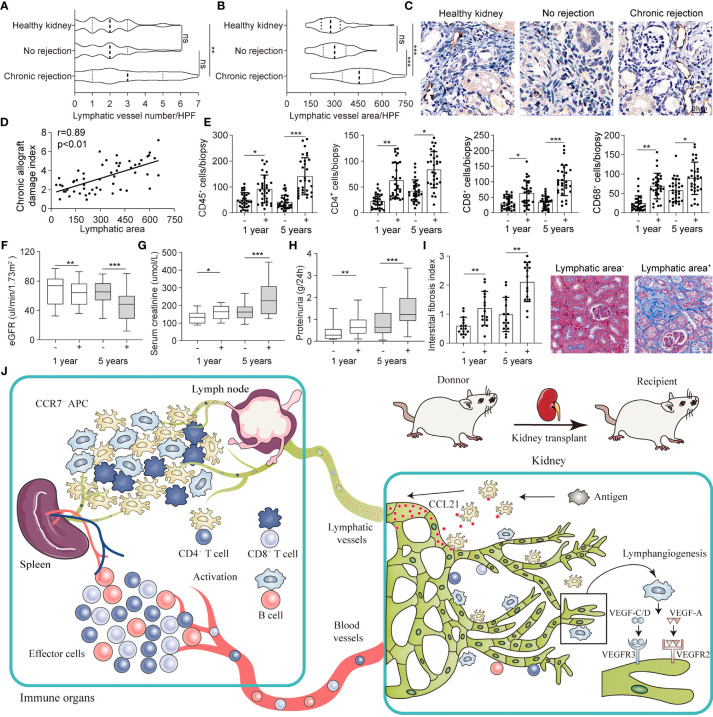
Intrarenal lymphangiogenesis negatively correlated with allograft function in transplant patients. **(A, B)** Lymphatic vessels number and area counted in high-power field (HPF) respectively in healthy (n=11), no rejection (n=80) and chronic rejection kidneys (n=54) from patients. **(C)** Representative images of immunohistochemical staining of LYVE-1 in healthy, no rejection and chronic rejection kidneys from patients to identify the situation of lymphangiogenesis. **(D)** Linear regression graph of chronic allograft damage index and lymphatic area. **(E)** Cell counting of different immune cells, include CD45^+^ (myeloid cells), CD4^+^ (Th cells), CD8^+^ (CTL cells), CD68^+^ (macrophage), in lymphatic area^+^ (defined as lymphatic vessels area more than 458.74 μm^2^) and lymphatic area^-^ (defined as lymphatic vessels area less than 458.74 μm^2^) group from chronic rejection kidneys at 1 year and 5 years determined by flow cytometry. **(F)** Quantitative data of eGFR (μL/min/1.73m^2^) in lymphatic area^+^ (n=36) and lymphatic area^-^ (n=18) group from chronic rejection kidneys at 1 year and 5 years. **(G)** Quantitative data of serum creatinine (μmol/L) in lymphatic area^+^ and lymphatic area^-^ group from chronic rejection kidneys at 1 year and 5 years. **(H)** Quantitative data of proteinuria (g/24h) in lymphatic area^+^ and lymphatic area^-^ group from chronic rejection kidneys at 1 year and 5 years. **(I)** Interstitial fibrosis index in lymphatic area^+^ and lymphatic area^-^ group from chronic rejection kidneys at 1 year and 5 years. Representative image of Masson staining showing the situation of interstitial fibrosis within lymphatic area^+^ and lymphatic area^-^ groups. **(J)** Schematic diagram concluding the crosstalk between kidney and immune organs in allograft rejection lymphangiogenesis. *P < 0.05, **P < 0.01, ***P < 0.001. Values are mean ± SEM.

To evaluate the function of lymphangiogenesis in human kidney transplantation, we conducted a cross-sectional analysis for the association of allograft rejection and renal lymphangiogenesis in a cohort of 145 patients with biopsy (11 healthy kidneys, 80 no rejection kidneys, and 54 chronic rejection kidneys respectively). It is revealed that the number density of LVs in chronic rejection group did not show any significant changes compared with healthy and no rejection patients, mirroring the LVs expansion pattern in heart transplant (**Figure 6A**) (20). Of note, the area of LVs directed at a highly significant upregulation with an increase over 1-fold in chronic rejection patients with a mean of 458.74 μm^2^ versus 183.32 μm^2^ and 178.61 μm^2^ per 50 HPF in comparison with healthy and no rejection patients respectively (**Figures 6B, C**). Besides, we found that lymphangiogenic growth factors VEGF-C, VEGF-D, and FGF-2 were highly expressed in renal tubular epithelial cells and some renal interstitial cells in allografts with chronic rejection (**Figure S11**). A partial correlation analysis was further performed to determine the linear correlation between lymphatic area and chronic allograft damage index and found a significant positive association, with the correlation coefficient r of 0.89 (**Figure 6D**). These data suggest that chronic rejection kidneys in patients after renal allograft were greatly related to the lymphatic area.

Following above findings, we divided chronic rejection patients into high lymphatic density and low lymphatic density groups by intrarenal LV area with a mean of 309.74 μm^2^ from **Figure 6B**. We noticed that intrarenal infiltrated effector and regulatory immune cells which defined as CD45^+^ myeloid cells, CD4^+^ Th cells, CD8^+^ CTL cells and CD68^+^ macrophage were generally increased at 1 and 5 years after transplant in the lymphatic area^+^ group (**Figure 6E**). In line, posttransplant patients in which renal biopsy exhibited higher area density of LVs presented with lower eGFR, severe serum creatinine and proteinuria, and greater intrarenal interstitial fibrosis which suggested a chronic decrease in renal function (**Figures 6F**–**I**). Meanwhile, submicroscopic typical pathological changes of rejection were observed in lymphatic area^+^ group, including early graft glomerular lesion (Cg1a), peritubular capillary basement membrane multilayer lesion (PTCML), glomerular basement membrane (GBM) thickening and foot process (FP) fusion (**Figure S12**). Together, all these results indicated that lymphangiogenesis in renal allograft mediated immune cell trafficking, connected allograft regional microenvironment with circulating immune system, and eventually led to chronic failure of the transplanted kidneys (**Figure 6J**).

## Discussion

Although the survival rates of renal transplantation have improved remarkedly, alloimmune responses accompanied by renal fibrosis, glomerulosclerosis, and tubular atrophy remain challenging (27). These unrecognized and persistent chronic alloimmune responses are regarded to be the main cause for chronic graft failure (28, 29). To date, previous studies reported that LVs contributed to regulating renal blood flow and blood pressure (30), even the immune regulatory functionality of lymph in kidneys (31). A rat kidney study suggests that lymphangiogenesis is associated with chronic renal allograft injury (32). Here, extensive LEC proliferation and expansion were observed both in the mouse models and the patients with chronic allograft rejection. Our model of renal chronic rejection revealed severe cellular infiltration distributed around LVs through which CCR7^+^ cells were recruited to the RDLN by CCR7-CCL21 interaction. Suppression of lymphangiogenesis inhibited systemic CCR7^+^ cells trafficking and chronic rejection progression. Likewise, renal allograft patients with higher LVs area density showed chronic function failure.

In the surgery of organ transplant, the surgeon would not connect the LVs of donor organ to the recipient, which means that the lymphatic network of the transplanted organ is spontaneously rebuilt by the recipient through some kinds of mechanisms (33, 34). The LVs from donor kidney have not reconnected to recipient in renal transplant, and this procedure of how they rebuild interrupted lymph drainage after surgery remains to be poorly understood (35). Current researches suggest that the cellular mechanism of lymphangiogenesis in transplant could involve the incorporation of circulating progenitors or the division of local preexisting endothelial cells (19, 21, 36). However, the role of progenitors is still controversial for the complex origin and limited study method. Other studies with opposing results concluded that newly generated LVs “sprout” from the preexisting local lymphatic network which was investigated in murine lung transplant (21, 36). We utilized LYVE-1 EGFP-Cre mice as donors to observe co-localization of LYVE-1 and EGFP, and found that the restoration of lymph drainage was derived from the LVs of heterogeneous sprouting. Despite observing the restoration of lymph drainage, the anatomical procedure of reconnection remains to be explored.

Although it is difficult to illustrate the concrete mechanism of adaptive and innate immunity between the newly generated LVs and renal transplant, increasing evidence points to lymphangiogenesis as a crucial factor related to immune infiltration after human kidney transplantation (18). Researchers have reported that LVs can potentially provide a drainage pathway for inflammatory cells, including activated DCs and lymphocytes expressing CCL21 receptor CCR7 (37, 38), migrating to the organs or lymph node expressing CCL21 (39–42). Based on above discoveries, we measured the expression level of CCL21 in renal transplant biopsy tissue samples, and found it prominent increased in LVs. We also found that increased CCR7^+^ cells which were mostly composed of DCs, together with some macrophages and lymphocytes were distributed around the renal LVs, corresponding to a previous study that proved host DCs playing an essential role in organ transplant rejection (43). Since we found the CCR7-CCL21 axis as a mediation built the relationship between LVs and alloimmune in kidney transplantation, we next try to investigate what functions they each play in the chronic rejection of renal allograft. In addition to the role of chemotactic recruitment of inflammatory cells, CCR7-CCL21 interaction has the function of immune activation as well (44, 45). Early in 1970, Pedersen et al. has proved that the renal lymph flow in sheep with allograft kidneys increased by 20- to 50-fold with numerous inflammatory cells infiltration (46). CCR7-CCL21 interaction has proved to be the bridge between newly generated LVs and chronic renal rejection. However, the relationship between lymphangiogenesis and CCL21-CCR7 interaction is not only unidirectional, but reciprocal. Marinkovic et al. has reported that the construction of lymphoid structures was not discovered but observed ectopically with CCL21 expressing in CCR7-deficient mice (47). To further identify this function, we investigated the consequence of LV suppression by genetic techniques (LYVE-1-Cre/iDTR mice) (26) and administration of blocking biological reagents (48). Both these two kinds of models showed reduced lymphangiogenesis and inflammatory infiltration. Given the much more attention gained on DSA mediated rejection, the decreased levels of DSA and germinal center (GC) B cells further identified the alleviated inflammatory rejection. Reduced infiltration of T cells and activated B cells corresponded to the suppressed lymphangiogenesis and antigen presentation (49, 50). All these results suggested that CCR7 is indispensable for lymphangiogenesis and CCR7-CCL21 interaction has a reciprocal relationship with alloimmunity (51, 52).

Our mouse models showed that attenuated alloimmune responses corresponded to suppressed lymphangiogenesis, with alleviated chronic rejection, allograft fibrosis and renal function. Meanwhile, the patient population of renal allografts showed that higher LVs area density was negatively correlated with allograft functions, not the number density of lymphatic vessels. Worth to mention that, both the lymphatic area and number reflected the density of LVs, but the higher density of lymphatic vessels does not necessarily mean higher lymphatic flow (53, 54). The increase in lymphatic area is due to the different diameters of LVs. The larger diameter of LVs can result in a decrease of drainage resistance, while the increase in the number of LVs does not significantly improve drainage efficiency. Thus, increased lymphatic drainage through increased lymphatic area is correlated with the chronic allograft damage index, not the number. The same result also demonstrated in a heart transplantation article, which showed the lymphatic flow index is correlating with lymphatic area, not the number density (20).

Although our study demonstrated the lymphangiogenesis could deteriorate the function of renal allografts, some previous studies reported that lymphangiogenesis could prolong the life span of recipients with better renal functions both in mouse and human kidney transplant, which is totally contrary to our findings (35, 55). In parallel, a clinical study revealed human biopsy studies demonstrated kidney transplants with higher perivascular lymphatic density had a reduced proportion with progression of interstitial fibrosis/tubular atrophy grade (56). These studies held the perspective that LVs give play to provide excurrent pathways for cellular infiltration, and thus prevent continuous alloimmune responses in renal allografts. However, there were still many studies demonstrating similar result to ours. Interruption of the lymphatic drainage is believed to be an essential factor in cardiac allograft failure by facilitating myocardial fibrosis after cardiac transplantation (57). Also, suppression of lymphangiogenesis inhibited systemic CCR7^+^ cell trafficking and chronic rejection progression in lung allograft rejection (58). All these researches are regarded that lymphangiogenesis is detrimental in allografts by motivating antigen presentation within draining lymph nodes and stimulating alloimmune responses. Therefore, lymphangiogenesis was considered as a double-edged sword for renal allograft patients (59, 60).

## Conclusion

In summary, we have successfully established a mice model of renal chronic rejection, with characteristic pathological features. We have demonstrated that the reconnection of LVs after their broken during transplantation contributes to the donor antigen presenting and lymph nodes activating. Meanwhile, our studies observed obvious lymphangiogenesis and a rebuilt of interrupted lymph draining one week after surgery, involving preexisting LECs from both the donor and recipient, which suggests a rebuilt novel alloantigen presenting pathway. These expanding LVs released CCL21 and recruited CCR7^+^ cells, mainly DCs, toward lymph nodes and spleen, resulting the adaptive response. This rejection could be relieved by LYVE-1 specific LVs knockout, or CCR7 migration inhibition. Moreover, in our retrospective analysis, posttransplant patients exhibiting higher area density of LVs presented with lower eGFR, severe serum creatinine and proteinuria, and greater intrarenal interstitial fibrosis indicating a chronic decrease in renal function. These findings identify a novel pathway of doner alloantigen presenting through restoring the broken lymph flow and add to our understanding of the complex regulation of chronic rejection.

## Methods

### Animals

Itgax-Cre-EGFP, LYVE-1-EGFP-Cre and ROSA26.iDTR were purchased from The Jackson Laboratory. C57BL/6 (H-2b) and Balb/c (H-2d) mice (6-8 weeks) were purchased from Charles River (Beijing, China). Selective expressions of EGFP in the nuclei of LECs were obtained using the LYVE-1-EGFP-Cre mice in a C57BL/6 background. LYVE-1-Cre/iDTR mice were generated with intercrossing LYVE-1-Cre mice with ROSA26.iDTR and ablation of LYVE-1^+^ LVs were generated by intravenous diphtheria toxin (Sigma-Aldrich, D0564) at 30ng/mouse/day with 5 consecutive days before renal transplantation. Animal breeding and all experimental procedures were approved by the animal protection and Research Advisory Committee of the First Affiliated Hospital, School of Medicine, Zhejiang University.

### Cell Culture

Human lymphatic endothelial cells (HLECs) were purchased from Procell as well as cultured in EBM-2 medium supplemented with hEGF, hydrocortisone, heparin, 10% FBS, ascorbic acid and VEGF at 37°C in 5% CO_2_ atmosphere. CD11c^+^ DCs were isolated from the spleen of Itgax-EGFP mice and cultured in complete RPMI 1640 medium supplemented with 10 ng/ml GM-CSF as well as 1 μg/ml LPS at 37°C in 5% CO_2_ atmosphere.

### Mouse Kidney Transplantation

Vascularized kidney transplantations were established in mice as previously described (61–63). Briefly, C57BL/6 (H-2b) recipient as well as Balb/c (H-2d) donor mice weighing 25-30g were anesthetized with 3% sevoflurane (1.2 minimal alveolar concentration) in oxygen (25, 64). The left renal artery and vein of recipients were ligated and the original kidney was taken. After ligating the corresponding lumbar branch, the aorta and vena cava were closed by tightening the ring. 5 ml of 7.5% heparin sulfate solution was injected into the vena cava of the donor systematically. The donor tissues involving the aorta, vena cava and ureter with bladder dome were resected. Continuous 10-0 nylon suture was used for end to side vascular anastomosis. Then, the donor ureter was passed through the left vas deferens and 9-0 nylon thread was used for open bladder anastomosis. Overall, the total ischemic time averaged 25 to 35 minutes. The contralateral nephrectomy was anesthetized with 3% sevoflurane on day 3 post-transplant, avoiding an initial period of acute renal failure (65, 66). The technical success rate was around 78% and the mean survival was 66 ± 7.10 days. For the isograft group, C57BL/6 (H-2b) recipients were given a kidney from a littermate.

### DCs-LECs Chemotaxis Assay

1 × 10^4^ HLECs together with 1 × 10^3^ CD11c^+^ DCs isolated from Itgax-Cre-EGFP mice were seeded with 48-multiwell plate in the EGM-2 medium, which was previously coated with matrix gel. After 6 hours, the micropores were washed three times with PBS and fixed with 4% PFA. Finally, the adhesion of CD11c^+^ DCs to HLECs capillary-like structure was visualized using a Leica Inverted DMi8 microscope.

### Gene Silencing *In Vitro*

In order to silence CCL21 in LECs through siRNA transfection, 5 × 10^5^ HLECs were seeded in 6-well plates as well as 30 nM of total siRNA were transfected with duplexes targeting CCL21 using Lipofectamine 3000 (Invitrogen) based on the instructions. After three days, the cells were harvested for further analysis.

*CCR7 intervention in vitro and in vivo:* For blocking of CCR7 in LECs *in vitro*, 5 × 10^5^ HLECs were seeded in 6-well plates and incubated with anti-CCR7 monoclonal antibody (Abcam) at a dose of 10 μg/mL for 6 days consecutively. For intervening CCR7 *in vivo*, monoclonal anti-mouse CCR7 antibody (R&D Systems) was injected into recipients (30 μg/mouse) by intravenous injection.

### Evans Blue Dye Injection

0.5% wt/vol of Evans Blue dye (Sigma-Aldrich) diluted in a total of five microliters PBS was injected into the renal capsule as well as upper and lower pole of renal parenchyma using a 34-gauge syringe. Also, the mice were kept for around 20 minutes to let the dye migrate by the LVs as well as reached the LNs of interest. Next, the recipient mouse was euthanized by cervical dislocation. The pattern of lymph drainage in renal allograft was monitored and the blue labeled RDLNs were then harvested separately.

### Injection of DCs Under the Renal Capsule

Spleens from Itgax-EGFP mice were collected in RPMI 1640 medium (serum-free) containing 2mg/ml collagenase type IV, and then digested in 37°C shaking incubator for 30 minutes. Digestion was stopped with cold buffer (PBS containing 0.1 mM EDTA and 2.5% FCS) and digested tissue was filtered using a 70 μm strainer. Then, cells were re-suspended in FACS buffer and CD11c^+^ DCs were sorted by BD Influx Cell Sorter. EGFP+ CD11c+ DCs (1×10^5^) in 50 μl PBS were injected under the renal capsule of recipient mouse at week 1, 4 and 8 after transplantation surgery. Renal samples and RDLNs were then harvested separately and fixed in 4% paraformaldehyde (PFA).

### Histology and Immunostainings

For continuous 4 μm thick sections of kidney, lymph nodes or spleen, the tissue samples were fixed overnight in 4% PFA at 4°C, dehydrated and then embedded in paraffin. To evaluate renal fibrosis, Masson trichrome staining was performed as described previously (63). For immunostainings, the paraffin sections were rehydrated for further antigen recovery in EDTA antigen retrieval buffer (pH 8.0) at 100°C for 20 minutes. For immunohistochemistry, 0.5% hydrogen peroxide was regularly added to methanol to block the activity of endogenous peroxidase. Furthermore, in order to block the nonspecific binding, 3% BSA was added for 1 hour. Then, slides was incubated with antibodies (diluted with PBS appropriately) overnight at 4°C with the following primary antibodies: VEGFR3 (1:100, Abcam), LYVE-1 (1:100, Abcam), CD31 (1:100, Abcam), VEGF-D (1:100, R&D Systems), VEGF-C (1:50, Abcam), F4-80 (1:100, Abcam), FGF-2 (1:100, R&D Systems), CD3 (1:100, Invitrogen), Ly6G (1:100, R&D Systems), CD19 (1:100, Abcam), CCR7 (1:50, R&D Systems), CD45 (1:50, Invitrogen), CCL21 (1:100, Abcam), AQP1 (1:100, R&D Systems), PODXL (1:100, R&D Systems), HBEGF/DTR (1:100, Abcam), EGFP (1:50, Invitrogen) α-SMA (1:100, Abcam) and collagen I (1:100, Abcam). Next, the slides were incubated with Alexa 488, Cyanine 3 or Cyanine 5 conjugated secondary antibodies (Abcam) (appropriately respond to primary antibody in species) for 50 minutes at room temperature as well as 4′,6-diamidino-2-phenylindole (DAPI) was utilized in order to stained nuclei. Imaging and microscopic analysis were acquired with a Zeiss LSM 880 confocal microscope and Image-Pro Plus software (Media Cybernetics, Rockville, MD, USA).

### qRT-PCR

Total RNA of renal and spleen tissues was extracted using the RNeasy kit (Qiagen) on the basis of the instructions of the kit manufacturer. The RNA extracted from 1μg was reverse transcribed into cDNA by GoScript Reverse Transcription Kit (Promega). qRT-PCR was carried out using TaqMan Gene Expression Master Mix (Invitrogen) as well as analyzed on a Light Cycler 480 (Roche) with indicated primers. Relative gene expression was analyzed by ΔΔCt method and the primers were described in **Supplementary Table 1**.

### Chronic Allograft Damage Index Evaluation

The chronic allograft damage index (CADI) was calculated following previous reports. In detail, we prepared histologic samples from each group. All the samples were coded and calculated blindly according to randomization and clinical status. Two pathologists were invited to review and rate these samples independently. And the CADI score was developed basing on the individual CADI parameter (i) interstitial inflammation, (ii) tubular atrophy, (iii) tubular fibrosis, (iv) mesangial matrix expansion, (v) glomerular sclerosis, and (vi) intimal proliferation. Each individual parameter was scored from 0 to 3. If the total CADI score arrived at independently by the two pathologists differed more than one SD from the mean, a consensus reading was performed.

### Transmission Electron Microscopy

Renal tissue was fixed with 4% PFA containing 1% glutaraldehyde and 0.1 mol/l cacodylate based buffer. Following multiple rinse cycles with double distilled water gently, the samples were dehydrated in acetone fractionation series. All sections were post-fixed with 1% OsO_4_ and embedded in OCT. Compound (Sakura Finetech). Ultrathin sectioning was performed with a LEICA EM UC7FC7, cutting to about 90 nm thickness. Then, the sections were stained with lead citrate as well as uranium acetate. TEM was performed with a Tecnai G2 SpiritBiotwin at 80 KV.

### Flow Cytometry

Kidneys, spleens and lymph nodes were collected at various indicated time points post transplantation and single-cell suspensions were prepared as previously described (67, 68). Briefly, kidney tissue was digested with collagenase type I (1 mg/ml) in RPMI 1640 for 20 min at 37°C. Spleen and lymph node tissues were minced and ground into single-cell suspensions, followed by filtered through a 70 μm strainer in FACS buffer (2% FBS in PBS). After red blood cell lysis and blocking Fcγ receptors with (BD Pharmingen, clone 2.4G2) mouse anti-CD16/CD32, 1×10^6^ cells were incubated for 15min with the following FACS antibodies in FACS buffer: Fixable Viability Stain 510 (BD Pharmingen), PerCP-Cy5.5 conjugated to anti-mouse CD31 (BioLegend, clone 390), FITC conjugated to anti-mouse Podoplanin (BioLegend, clone NC-08), PE conjugated to anti-mouse Ki67 (BioLegend, clone 11F6), PerCP conjugated to anti-mouse CD3e (BD Pharmingen, clone 145-2C11), PE-Cy7 conjugated to anti-mouse CD19 (BD Pharmingen, clone 1D3), FITC conjugated to anti-mouse CD4 (BD Pharmingen, clone H129.19), PE conjugated to anti-mouse CD11c (BD Pharmingen, clone HL3), APC-Cy7 conjugated to anti-mouse CD45 (BD Pharmingen, clone 30-F11), BV421 conjugated to anti-mouse CD11b (BD Pharmingen, clone M1/70), BV480 conjugated to anti-mouse NK1.1 (BD Pharmingen, clone PK136), PerCP-Cy5.5 conjugated to anti-mouse CD8a (BD Pharmingen, clone 53-6.7), APC conjugated to anti-mouse F4/80 (BD Pharmingen, clone T45-2342) and BV605 conjugated to anti-mouse Ly-6G (BD Pharmingen, clone 1A8). Data were collected by Aurora Flow Cytometry (Cytek) pre-gated by size and granularity based on forward and side scatter and analyzed with FlowJo v10.0.7 software (TreeStar).

### Patient Population

A total of 145 patients involving renal transplantation or trauma-induced nephrectomy in the First Affiliated Hospital, Zhejiang University between January 2013 and December 2020 were included in our analysis. Among these patients, we selected patients who were diagnosed as healthy (n = 11, referring to traumatic nephrectomy) or had no rejection (n=80, excluding acute rejection and primary or secondary kidney disease post transplantation) or had chronic allograft rejection (n=54, containing chronic active T cell mediated rejection or/and chronic active antibody mediated rejection or/and chronic rejection). The demographic as well as clinico-pathologic characteristics of the patients at baseline are presented in **Tables S2**, **S3**. For one thing, procedural biopsies were performed at 6 months and 2 years after renal transplantation in all patients. For another, if the patient’s serum creatinine is higher than or equal to 30% than the baseline within 1 month, or proteinuria is higher than or equal to 0.5 g/d, or though serum creatinine was less than 30% higher than baseline within 3 months, the cause was unknown, then a clinically targeted renal biopsy was performed. Exclusion criteria were as follows: combined with other solid organ transplantation, the presence of other forms of chronic kidney disease (CKD) such as glomerulonephritis (GN) or acute kidney injury (AKI), as well as lack of available clinical data. Renal pathologists blindly evaluated inflammation and fibrosis based on the clinical data of corresponding patients according to established pathology guidelines and Banff 2017.

### Biochemical Analysis

Serum creatinine, albumin levels as well as proteinuria were measured using an automatic biochemical analyzer (Roche, Germany). The glomerular filtration rate (GFR) was determined as previously described (69). Briefly, the transcutaneous light-emitting diodes were fixed to the depilated skin on the back of mice. 10 μl FITC-sinistrin (40 mg/mL in PBS) was administered in a smooth but rapid bolus using insulin syringe by tail vein. The eGFR was calculated according to the decrease of fluorescence intensity over time.

### Statistical Analysis

The data were presented as means ± standard deviations (SD) or median (interquartile range). Data analyzing was performed in the software of GraphPad Prism v8.3.0. In order to compare the variables between the two groups, two tailed unpaired t-test was used, or an ordinary analysis of variance (ANOVA) for multiple comparisons was used when among more than two measures were compared. The association between lymphatic vessel area and the chronic allograft damage index was analyzed by Partial correlations. All independent experiments were repeated three times. The probability value was regarded as statistical significance when the value of P < 0.05. The P values of posttest were as follows: *P < 0.05; **P < 0.01; ***P < 0.001.

## Data Availability Statement

The original contributions presented in the study are included in the article/**Supplementary Material**. Further inquiries can be directed to the corresponding author.

## Ethics Statement

The studies involving human participants were reviewed and approved by the Ethics Committee of the First Affiliated Hospital of Medical College of Zhejiang University. The patients/participants provided their written informed consent to participate in this study. The animal study was reviewed and approved by the animal protection and Research Advisory Committee of the First Affiliated Hospital, School of Medicine, Zhejiang University.

## Author Contributions

JL and JC contributed to the designing and conception of this study. JL, YC, HZ and HW performed the experiments. JL, XY, MT, and YC acquired and analysed data. All authors contributed to the article and approved the submitted version.

## Funding

This work is supported by the National Natural Science Foundation of China (81770752, 81970651).

## Conflict of Interest

The authors declare that the research was conducted in the absence of any commercial or financial relationships that could be construed as a potential conflict of interest.

## Publisher’s Note

All claims expressed in this article are solely those of the authors and do not necessarily represent those of their affiliated organizations, or those of the publisher, the editors and the reviewers. Any product that may be evaluated in this article, or claim that may be made by its manufacturer, is not guaranteed or endorsed by the publisher.
