# Corrigendum: Lymphatic reconstruction in kidney allograft aggravates chronic rejection by promoting alloantigen presentation

**DOI:** 10.3389/fimmu.2022.1071763

**Published:** 2022-11-24

**Authors:** Jinwen Lin, Ying Chen, Huijuan Zhu, Kai Cheng, Huiping Wang, Xianping Yu, Mengmeng Tang, Jianghua Chen

**Affiliations:** ^1^ Kidney Disease Center, The First Affiliated Hospital, College of Medicine, Zhejiang University, Hangzhou, China; ^2^ Department of Pathology, The First Affiliated Hospital, College of Medicine, Zhejiang University, Hangzhou, China; ^3^ Xiangya School of Medicine, Central South University, Changsha, China

**Keywords:** transplantation, chronic rejection, inflammation, lymphangiogenesis, allograft

In the published article, there was an error in **Figure 1D** as published. To reveal the expression of VEGF-D at week 4 in the allograft group, it had been accidentally replaced by the acquired image of the allograft group at week 8 when we used AI software to typeset for manuscript preparation. The corrected **Figure 1** and its caption are shown as below:

**Figure 1:**
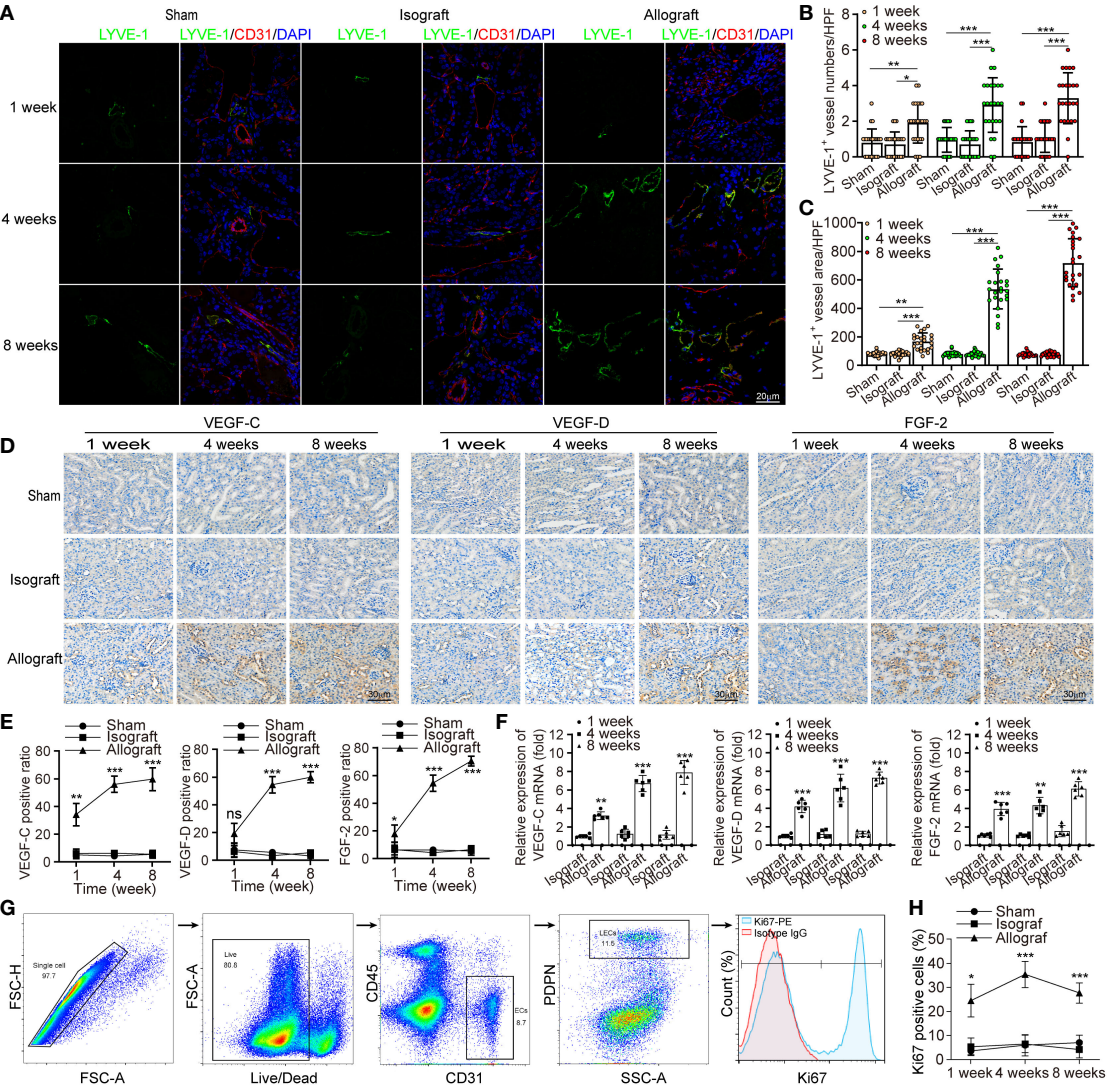
Chronic rejection is associated with lymphangiogenesis in renal allograft. **(A)** Representative immunofluorescence images of LYVE-1, CD31, and DAPI within sham (n=6 mice), isograft (n=6 mice) and allograft (n=6 mice) kidneys at 1, 4 and 8 weeks respectively. **(B, C)** Numbers and area counting of LYVE-1^+^ vessels in high-power field (HPF) at 1, 4 and 8 weeks respectively. **(D)** Immunohistochemistry of VEGF-C, VEGF-D and FGF-2 expression within sham, isograft and allograft kidneys at 1, 4 and 8 weeks respectively. **(E)** Positive ratio of VEGF-C, VEGF-D and FGF-2 within sham, isograft and allograft kidneys at 1, 4 and 8 weeks respectively. **(F)** Relative mRNA expression of VEGF-C, VEGF-D and FGF-2 by qRT-PCR within isograft and allograft kidneys at 1, 4 and 8 weeks respectively, using sham as a reference. **(G)** Live single CD45^-^ PDPN^+^ CD31^+^ LECs isolating from renal allograft by gating technology *via* flow cytometry, and the population of Ki67^+^ cells. **(H)** The ratio of Ki67^+^ cells in PDPN^+^CD31^+^ LECs within sham, isograft and allograft kidneys at 1, 4 and 8 weeks respectively. *P < 0.05, **P < 0.01, ***P < 0.001. Values are mean ± SEM.

The authors apologize for this error and state that this does not change the scientific conclusions of the article in any way. The original article has been updated.

## Publisher’s note

All claims expressed in this article are solely those of the authors and do not necessarily represent those of their affiliated organizations, or those of the publisher, the editors and the reviewers. Any product that may be evaluated in this article, or claim that may be made by its manufacturer, is not guaranteed or endorsed by the publisher.

