# Supplementary material for: Lymphatic Reconstruction in Kidney Allograft Aggravates Chronic Rejection by Promoting Alloantigen Presentation
Source: Front Immunol. 2021 Dec 9;12:796260. doi: 10.3389/fimmu.2021.796260 (PMC8695730; doi:10.3389/fimmu.2021.796260)
Supplement: Supplementary file 1 [file DataSheet_1.docx]

**SUPPLEMENTARY MATERIALS**

**Lymphatic reconstruction in kidney allograft aggravates chronic rejection by promoting alloantigen presentation**

**
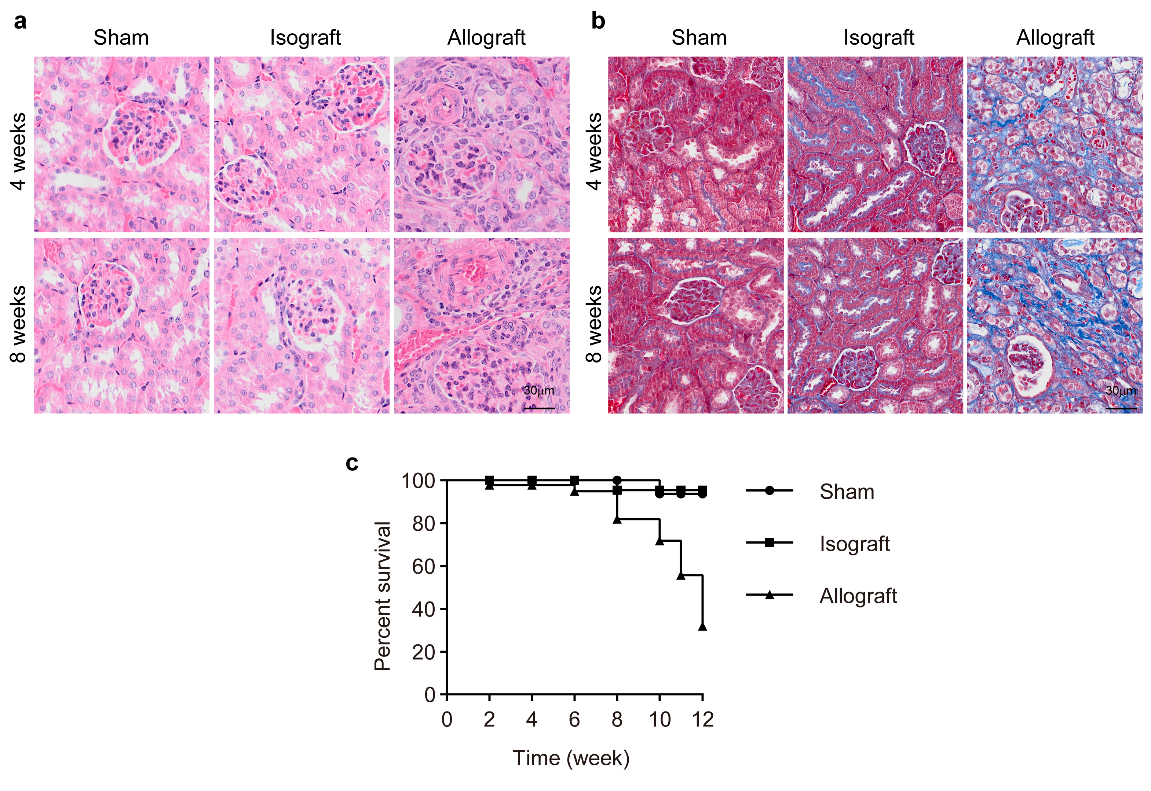
**

**Figure S1. Kidney allografts have histological features of chronic rejection.** (a) Haematoxylin and eosin staining of renal tissues at series time point post transplantation. (b) Masson staining of renal tissues at series time points post transplantation. (c) Precent survival of recipient mice in sham, isograft and allograft groups.

**
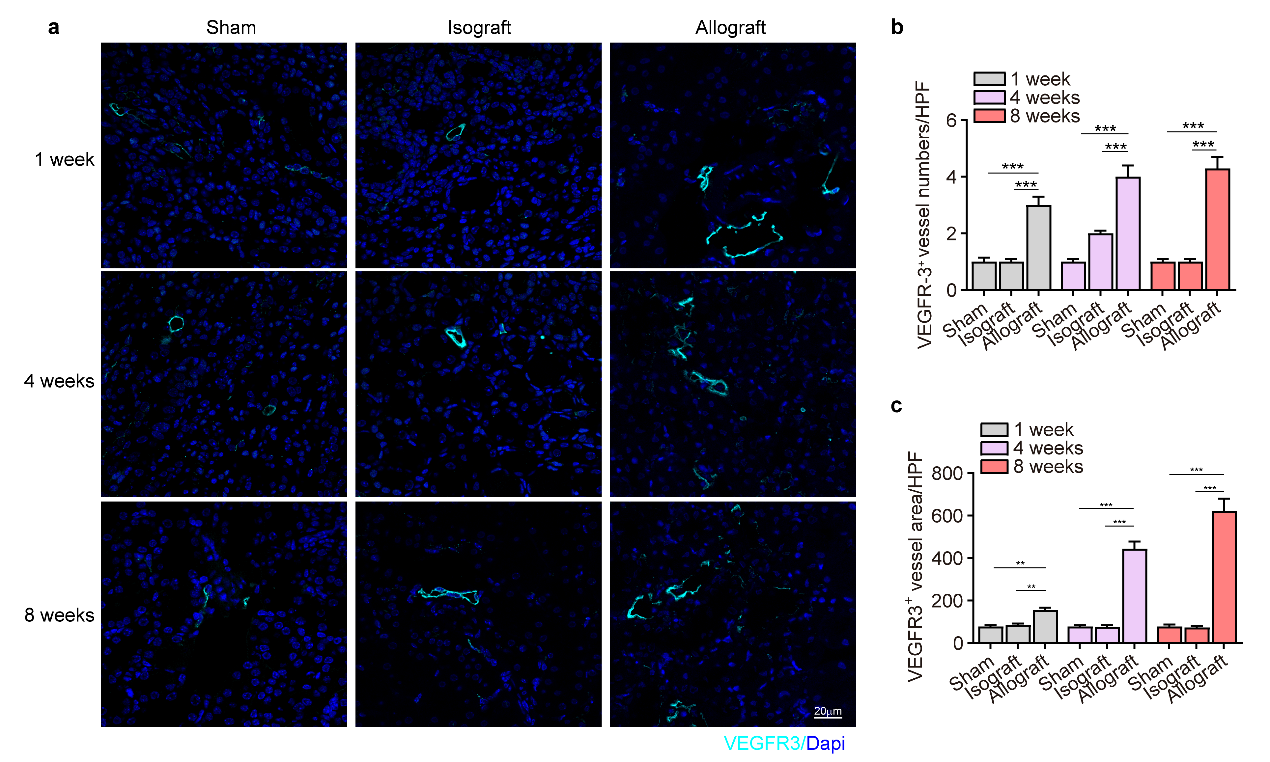
**

**Figure S2. Lymphangiogenesis observed through LVs marker VEGFR3.** (a) Representative immunofluorescence images of VEGFR3 within sham, isograft and allograft kidneys at 1, 4 and 8 weeks respectively. (b and c) Numbers and area counting of VEGFR3^+^ vessels in high-power field (HPF) at 1, 4 and 8 weeks respectively. *P < 0.05, **P < 0.01, ***P < 0.001. Values are mean ± SEM.

**
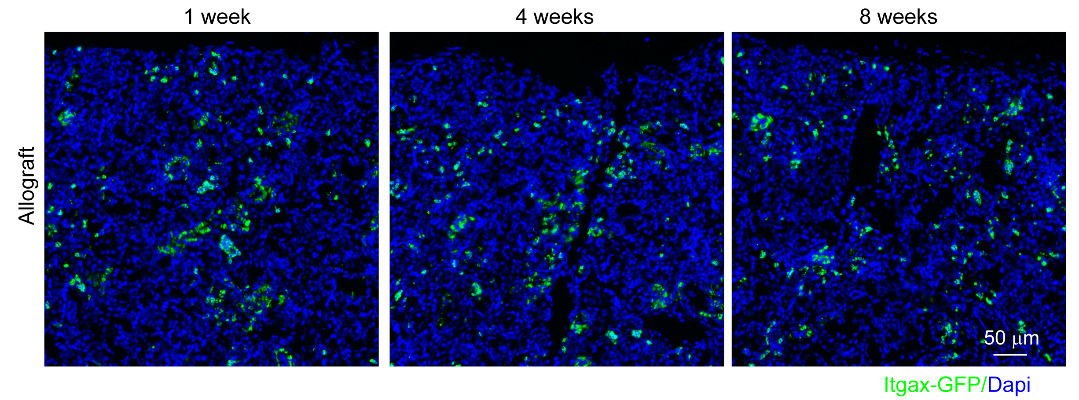
**

**Figure S3.** **Verify the presence of injected-dendritic cells in kidney.** GFP-expressing dendritic cells were isolated from peripheral blood of Itgax-Cre-GFP mice and injected under renal capsule. Their successful lodgment in kidney was then confirmed.


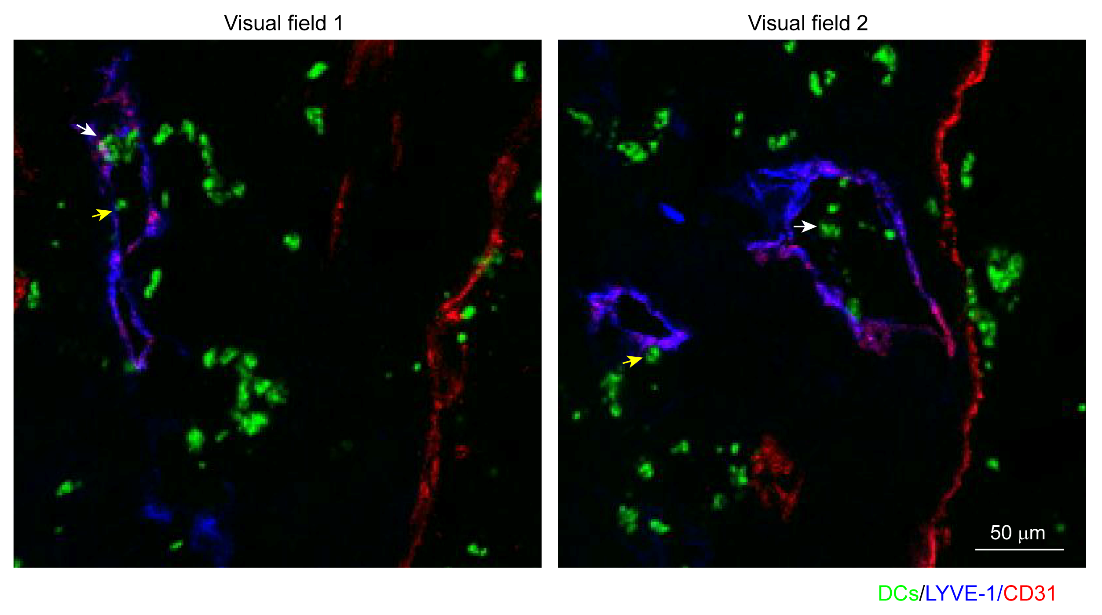


**Figure S4. Immunofluorescence analysis for DCs distribution.** Representative immunofluorescence images showed the location of DCs (green) in LYVE-1 marked (blue) lymphatic vessels and CD31 marked (red) vessels. White arrow presented injected DCs within the lymphatic vessels and yellow arrow presented injected DCs adhering the lymphatic wall.

**
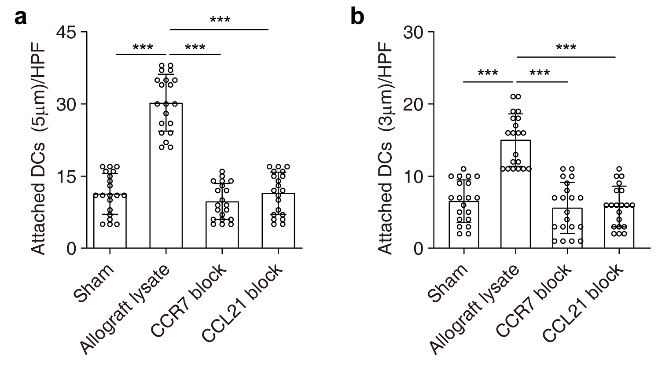
**

**Figure S5. Attached DCs number in tubule formation experiments.** (a) Number of attached DCs within 5μm of lymphatic capillary-like structures diameter per HPF field of view in sham, allograft lysate, CCR7 block and CCL21 block groups, respectively. (b) Number of attached DCs within 3μm of lymphatic capillary-like structures diameter per HPF field of view in sham, allograft lysate, CCR7 block and CCL21 block groups, respectively. ***P < 0.001. Values are mean ± SEM.


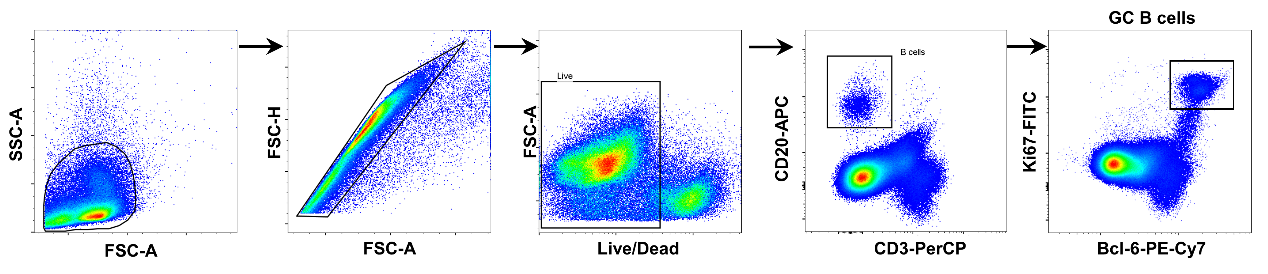


**Figure S6. Flow cytometry gating strategies.** Gating strategy for GC B cells used in Figure S7 and Figure S9. GC, germinal center.

**
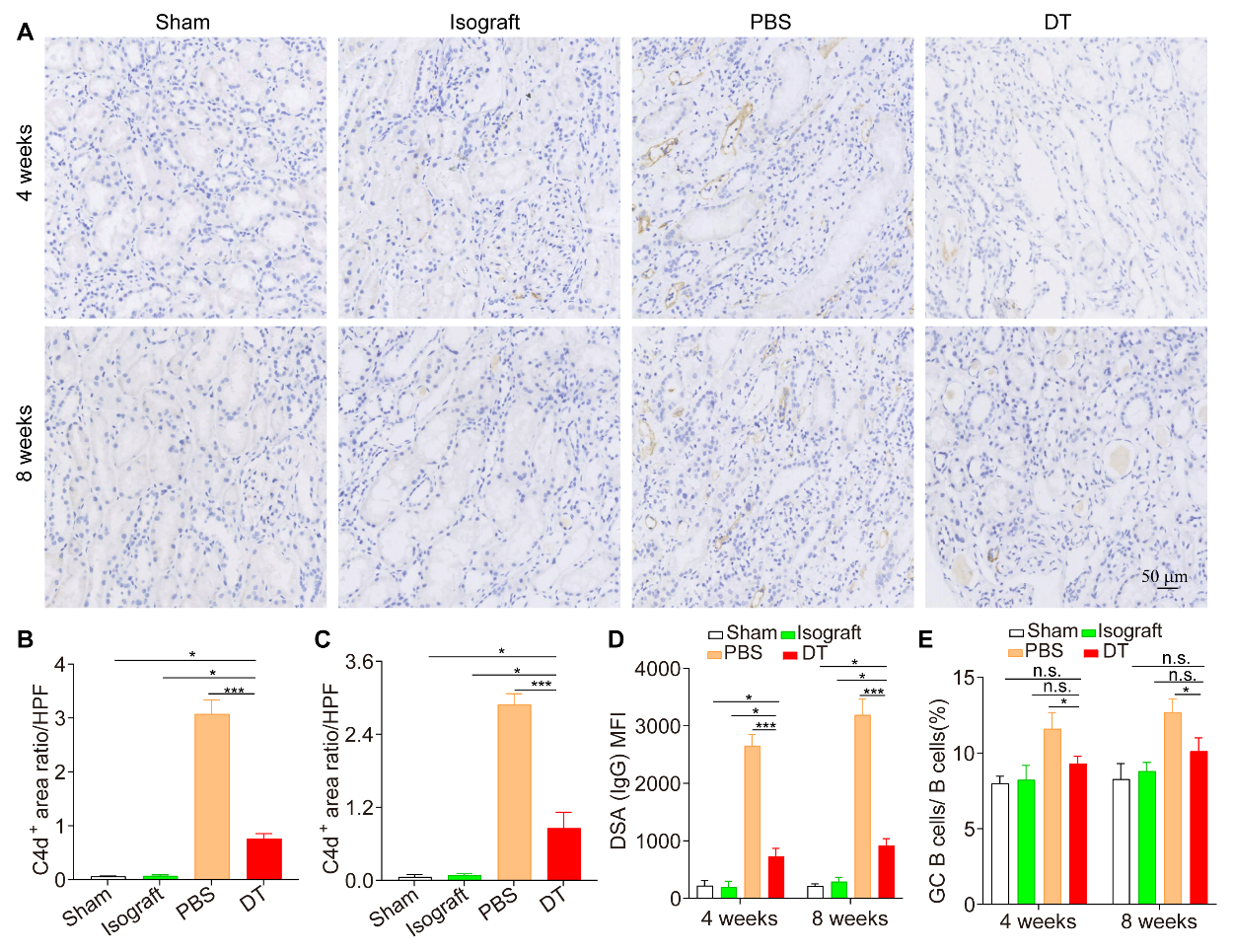
**

**Figure S7. Determination of C4d deposition, the levels of DSA as well as GC B cells after conditional knockout of LV with DT** (A) Immunohistochemistry for detecting C4d deposition in renal tissues at 4 weeks and 8 weeks in the groups of sham, isograft, PBS and DT treated, respectively. (B) Quantitative data of C4d^+^ area in different groups at 4 weeks post-transplantation. (C) Quantitative data of C4d^+^ area at 8 weeks post-transplantation. (D) The levels of DSA in peripheral blood in differently treated mice. (E) The percentage of GC B cells in relation to total B cells. DSA, donor-specific antibody; DT, diphtheria toxin; GC, germinal center. *P < 0.05, ***P < 0.001, n.s., not significant, Values are mean ± SEM.

**
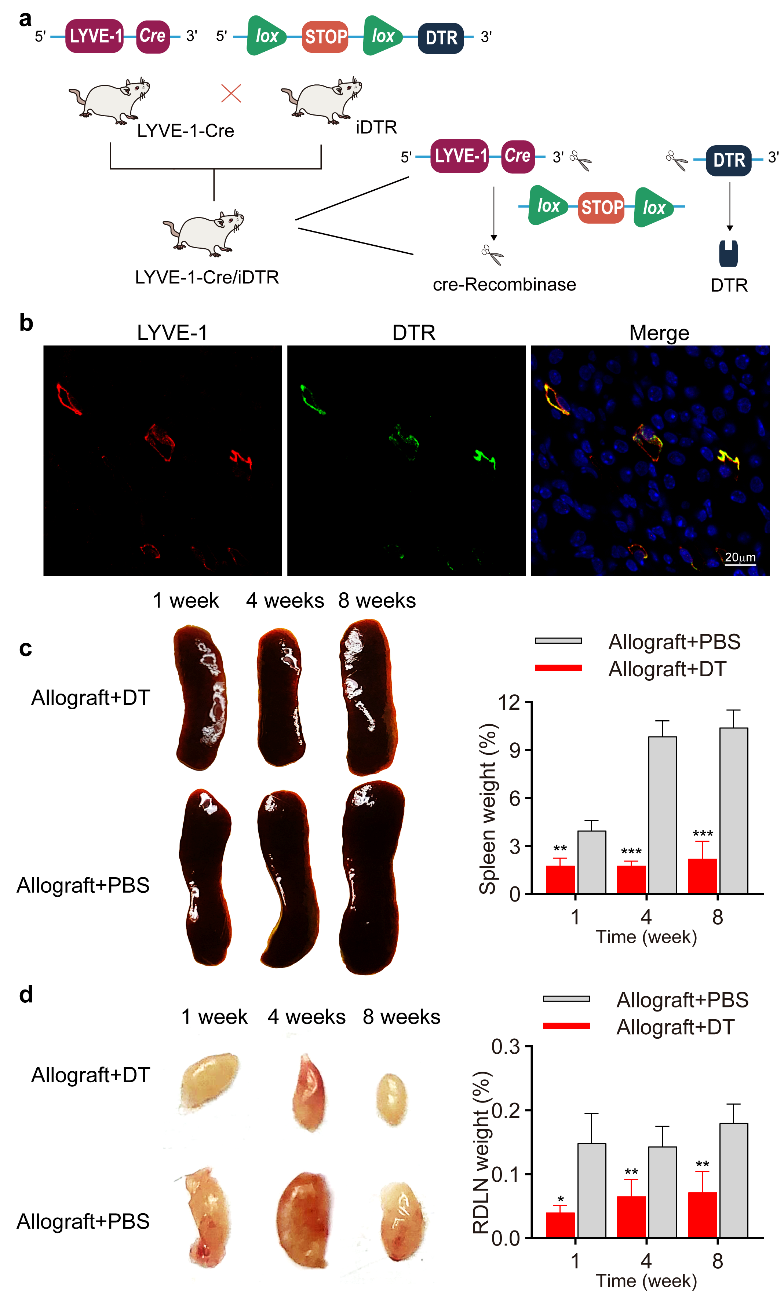
**

**Figure S8. Selective knockout of LVs in LYVE-1-Cre/iDTR mice attenuated splenomegaly and perirenal lymphadenectasis.** (a) Scheme showing the establishment of LYVE-1-Cre/iDTR mouse model. (b) LYVE-1 (red) and DTR (green) colocalization in the kidneys of LYVE-1-Cre/iDTR mice. (c and d) Changes of spleen and RDLN weight with DT administration at the 1, 4 and 8 weeks after surgery. *P < 0.05, **P < 0.01, ***P < 0.001. Values are mean ± SEM.

**
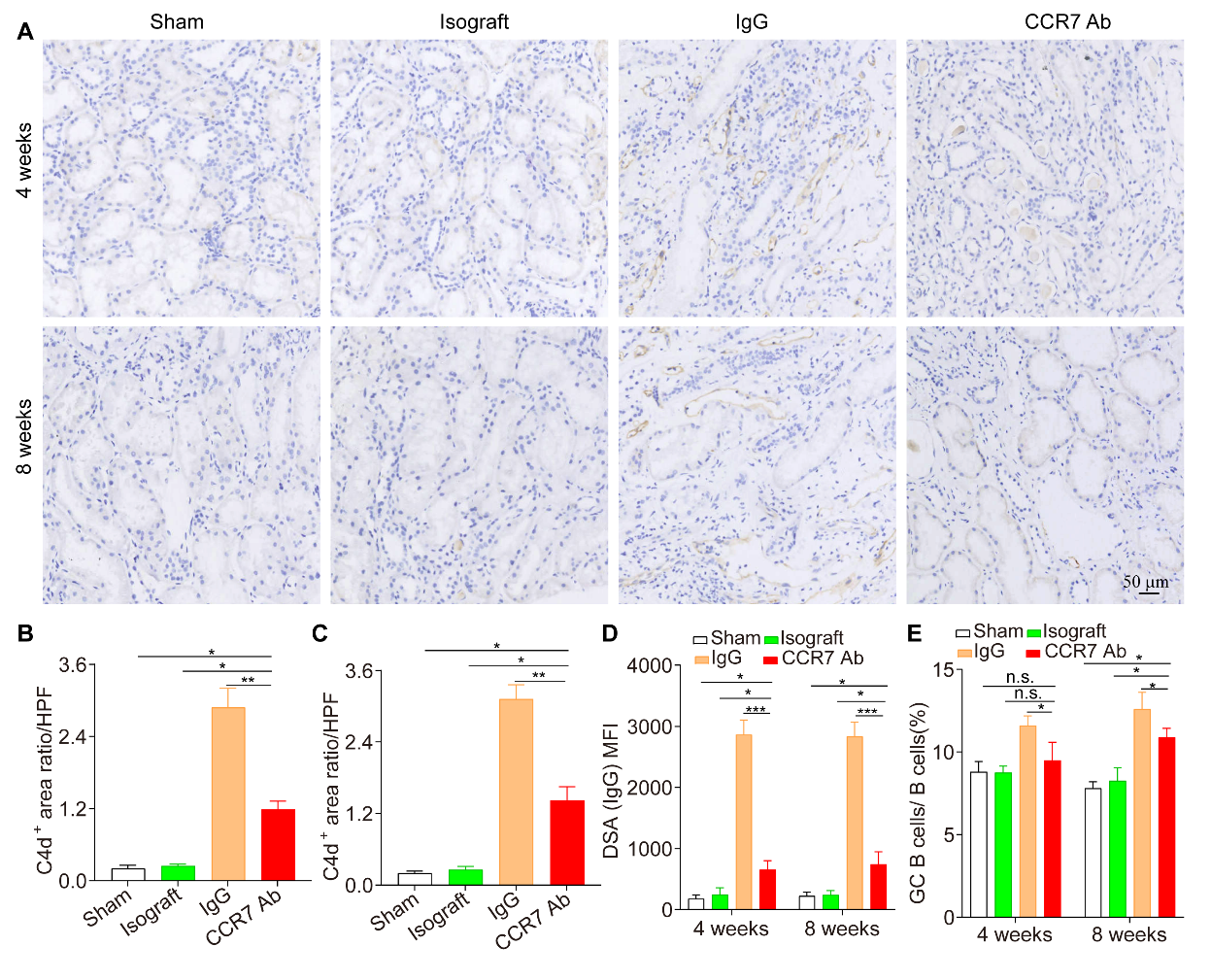
**

**Figure S9. The assessment of GC B cells and DSA-mediated rejection after CCR7 blocking antibody.** (A) Immunohistochemistry for C4d deposition in renal tissues at sham operation, isograft transplantation, IgG treated and CCR7 antibody treated groups, respectively. (B) Quantitative data of C4d^+^ area at 4 weeks after transplantation. (C) Quantitative data of C4d^+^ area at 8 weeks post-transplantation. (D) The levels of DSA in peripheral blood at 4 weeks and 8 weeks after treated, respectively. (E) The percentage of GC B cells in relation to total B cells. DSA, donor-specific antibody; GC, germinal center. *P < 0.05, **P < 0.01, ***P < 0.001, n.s., not significant, Values are mean ± SEM.

**
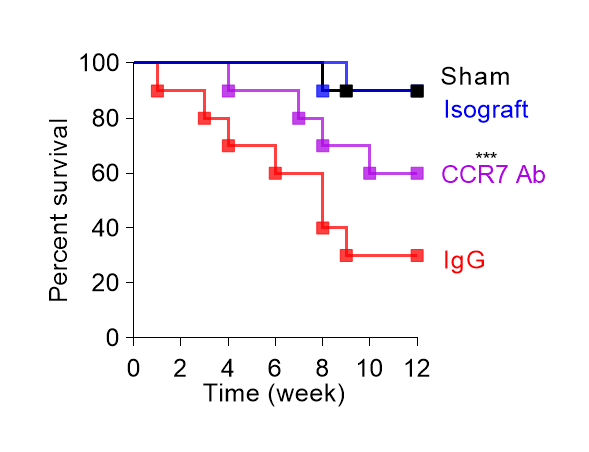
**

**Figure S10. Survival analysis of recipient mice.** The percent survival of recipient mice in the sham, CCR7 blocking antibody treated group and IgG treated group, respectively. ***P < 0.001.

**
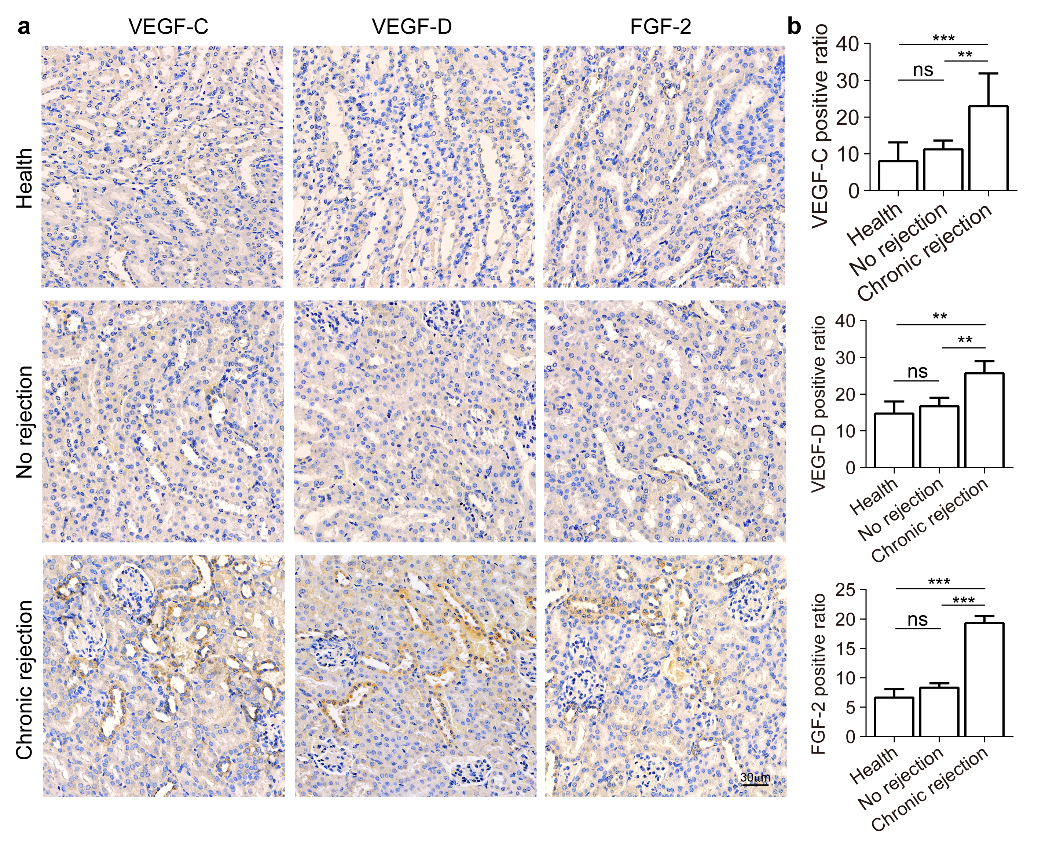
**

**Figure S11. Lymphangiogenic growth factors in kidneys of renal transplant patients.** (a) Immunohistochemistry of VEGF-C, VEGF-D and FGF-2 expression within health, no rejection and chronic rejection kidneys respectively. (b) Positive ratio of VEGF-C, VEGF-D and FGF-2 within health, no rejection and chronic rejection kidneys respectively. **P < 0.01, ***P < 0.001, n.s., not significant, Values are mean ± SEM.

**
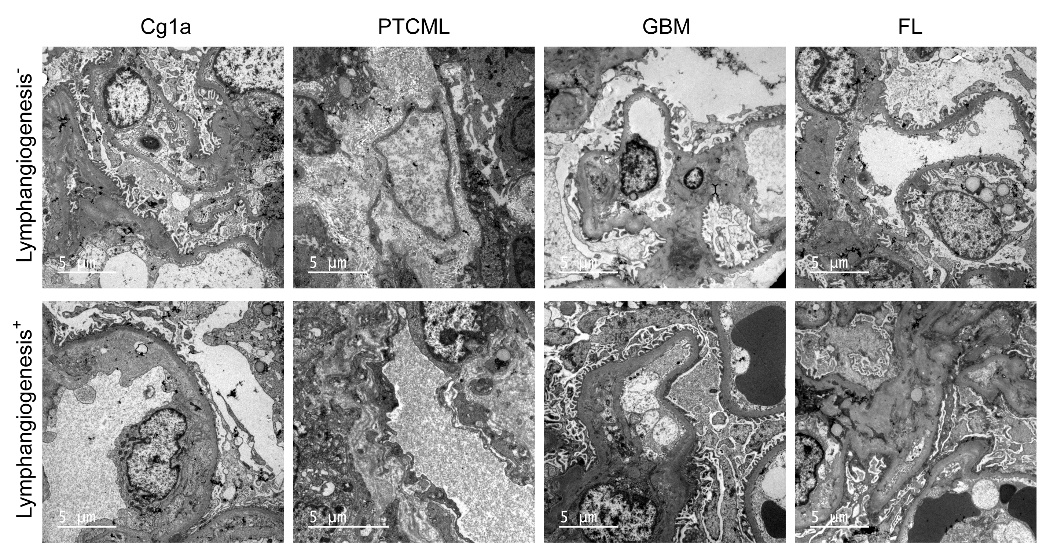
**

**Figure S12. Submicroscopic pathological changes of typical rejection.** Representative electron microscopy images of Cg1a, PTCML and changes in GBM and FP in lymphatic area^+^ and lymphatic area^-^ group respectively. Cg1a, early graft glomerular lesion; PTCML, peritubular capillary basement membrane multilayer lesion; GBM, glomerular basement membrane; FP, foot process.

**
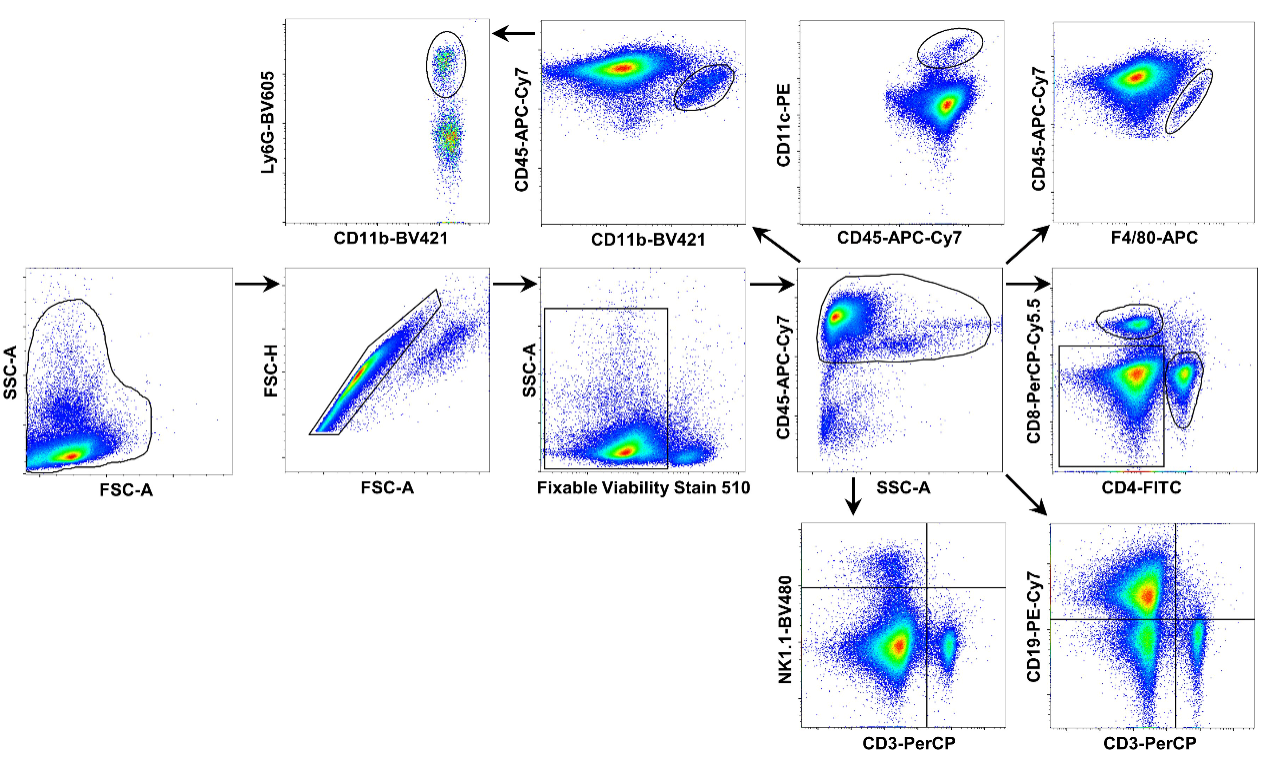
**

**Figure S13. Flow cytometry gating strategies.** Gating strategy used in Fig. 2E and I; Fig. 4E; Fig. 5G and I.

Table S1. Primers used for qRT-PCR.

| Abbreviation | Sequences | |
| --- | --- | --- |
|  | Forward | Reverse |
| VEGF-C | 5’GCCAATCACACTTCCTGCCGAT -3’ | 5’AGGTCTTGTTCGCTGCCTGACA -3’ |
| VEGF-D | 5′GACTGGAAGCTGTGGAGATGCA -3′ | 5′GGCTGCACTGAGTTCTTTGCCA -3′ |
| FGF-2 | 5’AGCGGCTGTACTGCAAAAACGG-3’ | 5'CCTTTGATAGACACAACTCCTCTC-3' |
| TNF | 5’GGTGCCTATGTCTCAGCCTCTT -3’ | 5’GCCATAGAACTGATGAGAGGGAG -3’ |
| IL1β | 5’TGCCACCTTTTGACAGTGATGA -3’ | 5’TGCCTGCCTGAAGCTCTTGT -3’ |
| IL2 | 5′AGAACTCAAACCTCTGGAGGAAG -3′ | 5′GCTGTCTCATCAGCATATTCACAC -3′ |
| IL4 | 5’CCGTAACAGACATCTTTGCTGCC -3’ | 5’GAGTGTCCTTCTCATGGTGGCT -3’ |
| IL6 | 5′AGACAGCCACTCACCTCTTCAG -3′ | 5′TTCTGCCAGTGCCTCTTTGCTG -3′ |
| IL10 | 5′TCTCCGAGATGCCTTCAGCAGA -3′ | 5′TCAGACAAGGCTTGGCAACCCA -3′ |
| IFNγ | 5′GAGTGTGGAGACCATCAAGGAAG -3′ | 5′TGCTTTGCGTTGGACATTCAAGTC -3′ |
| IL18 | 5′GATAGCCAGCCTAGAGGTATGG -3′ | 5′CCTTGATGTTATCAGGAGGATTCA -3′ |
| CCL7 | 5′ACAGAAGGACCACCAGTAGCCA-3′ | 5′GGTGCTTCATAAAGTCCTGGACC-3′ |
| CCL2 | 5′AGAATCACCAGCAGCAAGTGTCC-3′ | 5′TCCTGAACCCACTTCTGCTTGG-3′ |
| CCL3 | 5’ACTTTGAGACGAGCAGCCAGTG-3’ | 5’TTTCTGGACCCACTCCTCACTG-3’ |
| CCL5 | 5’CCTGCTGCTTTGCCTACATTGC-3’ | 5’ACACACTTGGCGGTTCTTTCGG-3’ |
| CCL8 | 5’TATCCAGAGGCTGGAGAGCTAC-3’ | 5’TGGAATCCCTGACCCATCTCTC-3’ |
| CCL9 | 5’TCCAGAGCAGTCTGAAGGCACA-3’ | 5’CCGTGAGTTATAGGACAGGCAG-3’ |
| CCL11 | 5’GCTACAGGAGAATCACCAGTGG-3’ | 5’GGAATCCTGCACCCACTTCTTC-3’ |
| CCL19 | 5’CGTGAGGAACTTCCACTACCTTC-3’ | 5’GTCTCTGGATGATGCGTTCTACC-3’ |
| CCL21 | 5′AGCAGGAACCAAGCTTAGGCTG-3′ | 5′GGTGTCTTGTCCAGATGCTGCA-3′ |
| CXCL1 | 5’AGCTTGCCTCAATCCTGCATCC-3’ | 5’TCCTTCAGGAACAGCCACCAGT-3’ |
| CXCL2 | 5’GGCAGAAAGCTTGTCTCAACCC-3’ | 5’CTCCTTCAGGAACAGCCACCAA-3’ |
| CXCL3 | 5′TTCACCTCAAGAACATCCAAAGTG-3′ | 5′TTCTTCCCATTCTTGAGTGTGGC-3′ |
| CXCL5 | 5′CAGACCACGCAAGGAGTTCATC-3′ | 5′TTCCTTCCCGTTCTTCAGGGAG-3′ |
| CXCL10 | 5′GGTGAGAAGAGATGTCTGAATCC-3′ | 5′GTCCATCCTTGGAAGCACTGCA-3′ |
| CXCL11 | 5′AAGGACAACGATGCCTAAATCCC-3′ | 5′CAGATGCCCTTTTCCAGGACTTC-3′ |
| CXCL12 | 5′CTCAACACTCCAAACTGTGCCC-3′ | 5′CTCCAGGTACTCCTGAATCCAC-3′ |

Table S2. Baseline demographic and clinic-pathologic characteristics of patients.

| Variable | Patients with Non- Rejection (n=80) | Patients with Chronic Rejection (n=54) | P-value |
| --- | --- | --- | --- |
| Age (years, mean ± SD) | 51.11±6.24 | 48.90±4.62 | 0.028 |
| Men, n (%) | 52(65.00) | 36(66.67) | NS |
| Re-transplantation | 0(0) | 1(1.85) | NS |
| DSA positive, n (%) | 0(0) | 46(85.18) | <0.001 |
| Creatinine (mmol/L) | 106.6±9.30 | 286.3±12.21 | <0.001 |
| Proteinuria (g/d) | 0.06±0.01 | 1.32±0.72 | <0.001 |
| eGFR (ml/min per 1.73 m^2^) | 78.6±6.94 | 36.8±4.21 | <0.001 |
| Immunosuppressive regimens, n (%) |  |  |  |
| Pred+ Tac+MMF | 54(67.5) | 31(57.40) | NS |
| Pred+CyA+MMF | 21(26.25) | 17(31.48) | NS |
| Pred+CyA+Aza | 5(6.25) | 4(7.40) | NS |
| Pred+CyA | 0(0) | 2(3.70) | NS |
| Biopsy Banff Scores (mean ± SD) |  |  |  |
| Glomerulitis (g) | 0.00±0.00 | 1.21±0.53 | <0.001 |
| Peritubular capillaritis (ptc) | 0.00±0.00 | 1.49±0.89 | <0.001 |
| Tubulitis (t) | 0.00±0.00 | 0.84±0.39 | <0.001 |
| Interstitial infiltrate (i) | 0.00±0.00 | 1.61±0.62 | <0.001 |
| Intimal arteritis (v) | 0.00±0.00 | 0.67±0.11 | <0.001 |
| Allograft glomerulopathy (cg) | 0.00±0.00 | 0.89±0.66 | <0.001 |
| Interstitial fibrosis (ci) | 0.00±0.00 | 1.60±0.92 | <0.001 |
| Tubular atrophy (ct) | 0.00±0.00 | 1.38±0.68 | <0.001 |
| Vascular fibrous intimal thickening (cv) | 0.00±0.00 | 0.82±0.41 | <0.001 |
| C4d+ n (%) | 0 (0) | 40 (75.92) | <0.001 |
| Follow up (month, mean ± SD) | 69.90±4.20 | 71.30±8.61 | NS |

NS, no significance; DSA, donor-specific antibody; eGFR, estimated glomerular filtration rate; Pred, prednisolone; Aza, azathioprine; Tac, tacrolimus; MMF, mycophenolate mofetil.

Table S3. Baseline characteristics at the time of transplantation.

| Variable |  | | Patients with Non- Rejection (n=80) | | Patients with Chronic Rejection (n=54) | P-value |
| --- | --- | --- | --- | --- | --- | --- |
| Recipient | |  | |  |  |  |
| Men, n (%) | |  | | 52(65.00) | 36(66.67) | NS |
| Age (yr) | |  | | 51.11±6.24 | 48.9±4.62 | 0.028 |
| BMI (kg/m2), mean ± SD | |  | | 22.3±3.9 | 21.6±3.5 | NS |
| Re-transplantation | |  | | 0(0) | 1(1.85) | NS |
| Time since dialysis (m) | |  | | 18.5±16.5 | 15.8±17.2 | NS |
| Recipient blood type – n (%)  A  B  AB  O | |  | | 18(22.50)  12(15.00)  10(12.50)  40(50.00) | 8(14.81)  3(3.56)  10(18.51)  33(61.11) |  |
|  |  |  |  |  |  |  |
|  |  |  |  |  |  | NS |
|  |  |  |  |  |  | NS |
|  |  |  |  |  |  | NS |
|  |  |  |  |  |  | NS |
| Donor | |  | |  |  |  |
| Men, n (%) | |  | | 46(60.00) | 37(68.52) | NS |
| Age (yr) | |  | | 36.2±10.50 | 33.4±8.10 | NS |
| Deceased, n (%) | |  | | 58(72.50) | 42(77.78) | NS |
| Cold ischemic time (min) | |  | | 785±305 | 812±346 | NS |

NS, no significance.
